# Examining the Triple Code Model in numerical cognition: An fMRI study

**DOI:** 10.1371/journal.pone.0199247

**Published:** 2018-06-28

**Authors:** Mikael Skagenholt, Ulf Träff, Daniel Västfjäll, Kenny Skagerlund

**Affiliations:** 1 Department of Behavioural Sciences and Learning, Linköping University, Linköping, Sweden; 2 Department of Management and Engineering, Division of Economics, JEDI-Lab, Linköping University, Linköping, Sweden; 3 Decision Research, Eugene, OR, United States of America; 4 Department of Psychology, University of Oregon, Eugene, OR, United States of America; 5 Center for Social and Affective Neuroscience (CSAN), Linköping University, Linköping, Sweden; University of California, San Francisco, UNITED STATES

## Abstract

The Triple Code Model (TCM) of numerical cognition argues for the existence of three representational codes for number: Arabic digits, verbal number words, and analog nonsymbolic magnitude representations, each subserved by functionally dissociated neural substrates. Despite the popularity of the TCM, no study to date has explored all three numerical codes within one fMRI paradigm. We administered three tasks, associated with each of the aforementioned numerical codes, in order to explore the neural correlates of numerosity processing in a sample of adults (*N* = 46). Independent task–control contrast analyses revealed task-dependent activity in partial support of the model, but also highlight the inherent complexity of a distributed and overlapping fronto-parietal network involved in all numerical codes. The results indicate that the TCM correctly predicts the existence of some functionally dissociated neural substrates, but requires an update that accounts for interactions with attentional processes. Parametric contrasts corresponding to differences in task difficulty revealed specific neural correlates of the distance effect, where closely spaced numbers become more difficult to discriminate than numbers spaced further apart. A conjunction analysis illustrated overlapping neural correlates across all tasks, in line with recent proposals for a fronto-parietal network of number processing. We additionally provide tentative results suggesting the involvement of format-independent numerosity-sensitive retinotopic maps in the early visual stream, extending previous findings of nonsymbolic stimulus selectivity. We discuss the functional roles of the components associated with the model, as well as the purported fronto-parietal network, and offer arguments in favor of revising the TCM.

## Introduction

Numbers are pervasive features of daily life, perceived and manipulated in activities such as trade and shopping, living within economic means, timekeeping, and successful communication of statistics, weights, and distances. Poor numeracy skills have been observed to negatively influence mental well-being and employment rates to a larger extent than poor literacy [1], and additionally incur extensive socio-economic costs related to special resources for areas such as education, employment, crime, and healthcare [2]. These concerns highlight the importance of an elementary knowledge of mathematics and a basic level of numerical competence for life in modern society. Although considerable research effort has been devoted to the understanding of numerical cognition, the developmental factors and neural mechanisms associated with both typical and atypical number processing are still unresolved issues (e.g. [3]). The leading Triple Code Model (TCM) of numerical cognition [4] argues for the existence of three primary representational codes for number, subserved by functionally distinct neural substrates: the visual Arabic number form (e.g. “3”), the auditory verbal word frame (e.g. “three”), and analog nonsymbolic magnitude representations (e.g. •••) [4]. Despite the popularity of the TCM [5], no study with adult participants (but see [6] for a study of the TCM in 9–12 year old children) has to date investigated the neurocognitive foundations of all three representational codes within one functional Magnetic Resonance Imaging (fMRI) paradigm (see [7] for a first structural connectivity account of the model). Previous research has primarily focused on the analysis of up to two concurrent codes [8], providing only a partial overview of the entire model. In order to bridge this important gap in the literature on numerical cognition, this study aims to provide experimental evidence examining the neural substrates of visual, verbal, and nonsymbolic numerical representation in line with the TCM.

Humans and some non-human animals, such as monkeys, pigeons, rats [9], and even invertebrates lacking complex cortical structures (such as spiders and honeybees [10]) appear to be endowed with a phylogenetically shared and innate capacity to perceive and manipulate analog nonsymbolic magnitude representations in an approximate manner [11–16]. This preverbal *Approximate Number System* (ANS) is crucial for the ability to estimate and represent the numerosity of, for instance, the number of wild animals in a pack or the tree bearing the largest amount of fruit, which informs decisions to hunt and gather efficiently. The precision of numerosity estimation, often referred to as ANS acuity, has been shown to predict mathematical abilities during development and is hypothesized to underlie the development of arithmetical and mathematical capacities [13, 17]. Over the course of typical development, the discriminability of numerical quantities becomes increasingly fine-grained as a result of mathematical education [14] and biological maturation [18]. As young children develop numerical language skills and a language-based symbolic number system, there is reason to believe that verbal number words and visually perceived Arabic digits are mapped onto the ANS [12], which has been argued to act as the primary semantic representation of numbers [4]. During this process of symbolic number acquisition, typically developing children begin to understand numerical cardinality as a linear concept (i.e. that a number *N+1* has a value of exactly *one more* than *N*), which transforms the conception of numerical distance from a compressed and logarithmic model towards an evenly spaced understanding of numerical distance [14, 19].

The intraparietal sulcus (IPS) has consistently been identified as the primary neural correlate of the ANS [20–22]. Research focusing on the neural substrates of numerical processing has now formed a broad consensus for the hypothesis that neuronal populations in the IPS support the representation of quantity, either in the form of an analog *more–less* magnitude code [15] or as a discrete representation that encodes cardinality [23]. The IPS is furthermore argued to be the primary region to process numerical information [5], acting as a semantic decoder of symbolic numerical input (e.g. Arabic digits, verbal number words) from domain-specific numerical systems [4] and active during numerical comparative operations [24]. This comparative operation is subject to a so-called *distance effect* [25], where the discrimination of two numbers spaced far apart (e.g. 1 versus 9) has reliably been shown to be easier than for numbers spaced closely together (e.g. 5 versus 6) [26]. These findings suggest that numerosity is represented in the form of a *mental number line*, where numerically similar values occupy approximately the same position on the numerical continuum; hence impeding the retrieval of appropriate representations for closely spaced numbers [26, 27]. The effects of development and symbolic mathematical education are especially apparent when young children are asked to place cardinal numbers along a horizontal line, where the distance between small numbers tends to be greatly inflated in comparison to tightly compressed larger numbers [19]. This behavior significantly differs from placement strategies found in older children and adolescents, who—aided by symbolic numerical knowledge—consistently demonstrate more linear conceptions of the mental number line [14].

A few recent studies have begun to challenge the dominant assumption of format-independent numerical representation in the IPS. Bulthé, De Smedt, and Op de Beeck [28, 29] used multi-voxel pattern analysis to demonstrate a lack of overlapping activity patterns for symbolic and nonsymbolic representational formats in this region. The authors found that Arabic digits and dots, matched on cardinality, elicited no unique activity patterns compared to unmatched cardinalities, which entails a need for principled consideration of exclusively format-dependent neural correlates of numerosity processing. One such recent alternative could be found in visual number processing, arguing that nonsymbolic magnitude processing begins in the early visual stream, in line with evidence of numerosity-sensitive retinotopic maps in areas V2 and V3 [30–32]. In this vein, Park et al. [33] argue that the human visual system is functionally predisposed to detect both general numerosity and changes in numerosity, with a sensitivity exceeding that of most other visual properties (cf. [34]). While such an account deviates from the original conception of the TCM, these results indicate the possible existence of low-level, asemantic perceptual neural substrates of nonsymbolic numerical cognition.

The combination of verbally supported symbolic number knowledge and preverbal nonsymbolic magnitude representations make up the foundation of typical numeracy skills, as reflected in the Triple Code Model [4, 5, 24, 35, 36]. Previous research in line with the TCM has identified dissociations corresponding to the three numerical formats, suggesting the functional and neurological diversity of these codes. The case for dissociation between nonsymbolic and verbal numerical representation was examined by Jefferies, Bateman, and Lambon Ralph [37], who argued that a patient suffering from semantic dementia (as a result of a left temporal lobe atrophy) could still proficiently judge the relative magnitude of Arabic numerals and dot arrays. However, the patient’s verbal production and perception of numerical language was impaired, suggesting separate functional substrates for the three codes. Similar results have been obtained for impaired visual Arabic numerical representation, while sparing both nonsymbolic and verbal numerical representations [38]. The fact that young children learn to associate verbal number words with quantities well before understanding written Arabic notations [39] similarly suggests that the verbal and visual codes are functionally distinct. Such clear dissociations are far from the norm, as seen in Dehaene’s [15] patient Mr. M, who suffered a right inferior parietal cortex lesion. Interestingly, Mr. M was able to effortlessly read number words and Arabic numerals, but had largely lost the ability to compare and discriminate numerical size, had severe difficulties in establishing the most proximate number to a given numeral, could not perform addition or subtraction tasks above chance level, yet was able to perform arithmetic operations on concrete nonsymbolic magnitude stimuli; such as dividing marbles between fictional characters. These examples of complex dissociations between symbolic and nonsymbolic numeracy skills, some of which implicate arithmetical operations, suggest a need for further research into the behavioral characteristics and neural substrates of the Triple Code Model.

Recent studies increasingly argue for the existence of a functionally distinct neural correlate of the visual Arabic code: the *Visual Number Form Area* (VNFA) [40]. Although the specific location and existence of the VNFA is debated [36], intracranial electroencephalography (EEG) [40] and fMRI-based [41] evidence suggests that the right caudal fusiform gyrus is exclusively responsive to Arabic numerals. A recent fMRI meta-analysis by Yeo et al. [42] indicates at least five candidate areas of the VNFA, spanning inferior and superior portions of the inferior temporal gyrus (ITG). These discrepant results may partially be attributed to common fMRI signal dropout in portions of the ventral occipito-temporal cortex and the ITG [40, 43]. Price and Ansari [36] found that passive viewing of Arabic digits elicited activation in the left ventral angular gyrus (AG), arguing this location to be a more relevant neural correlate of visual number processing than a purported ventral-stream VNFA. A consistent location of the VNFA has therefore not been established to date, although most recent evidence largely converges on the right ITG as a key area for visual numerical processing [42]. While recent studies increasingly suggest the involvement of multiple domain-general areas during Arabic number processing [22, 44], the frequent use of Arabic digits may support the development of a domain-specific functional region for such stimuli in ontogeny (cf. [42]); akin to the existence of a visual word form area despite the evolutionary recency of written communication [45, 46]. The modular assumptions of the TCM specifically implicate the separation and functional dedication of the Arabic code [4], and while this approach may be outdated in comparison to recent evidence [44], we find it important to closely adhere to the predictions of the model when evaluating its neural substrates.

Neural correlates of the verbal number word code have primarily been identified in left-hemispheric language areas. Schmithorst and Douglas Brown [35] provided evidence of the verbal code in the inferior frontal gyrus (IFG), and throughout the perisylvian language network: including the supramarginal gyrus (SMG), middle temporal gyrus (MTG), and superior temporal gyrus (STG). Dehaene et al. [24] furthermore identified the left AG as a central component of the verbal number word code, given the frequency of findings connecting AG to general verbal processing and verbal memory; especially arithmetic facts such as rote multiplication. In Dehaene and Cohen’s [47] account of the Triple Code Model, two processing routes of verbal number words were suggested: a direct route accessing rote verbal arithmetic knowledge, primarily implicating the left AG and the left cortico-subcortical loop (cf. [24]), and an indirect route used when rote memory is unavailable (e.g. in subtraction problems). The indirect route, in line with Schmithorst and Douglas Brown [35], primarily targets the perisylvian language network and the inferior parietal cortex. In a recent structural investigation of the Triple Code Model, Klein et al. [7] found a verbally mediated numerical pathway extending from the retro-splenial cortex to the ventromedial prefrontal cortex (VMPFC) through the ventral stream, with structural connections to the AG, MTG, and hippocampus. These findings suggest that the neural correlates of the verbal number word code are consistent in the literature, with areas of interest primarily limited to the left AG and left perisylvian language network, IFG, as well as the VMPFC.

The IPS has been tied to the processing of comparative magnitude judgments in domains outside of numerical cognition, such as spatial and temporal processing [48], suggesting the existence of a shared and partially overlapping network for general magnitude and numerosity processing [49]. In this vein, it appears likely that numerical cognition is subserved by a fronto-parietal network that minimally implicates the IPS, superior parietal lobule (SPL), superior frontal gyrus (SFG), middle frontal gyrus (MFG), supplementary motor area (SMA), and anterior cingulate cortex (ACC) [44, 50]. Such results challenge the previously predominant focus on the parietal lobes and the IPS in numerical cognition [51, 52], and suggests a need for systematic investigation of the neural correlates associated with the Triple Code Model. To this end, we developed an fMRI paradigm including tasks relevant to all three numerical codes. A whole-brain voxel-wise analysis was performed to investigate how the brain processes numerical information and performs numerosity discrimination for Arabic numbers, verbal number words, and analog nonsymbolic dot arrays. A conjunction analysis was then performed in order to investigate whether the purported fronto-parietal network underlying numerical cognition [44] was tapped in all experimental tasks, and the extent to which certain functional areas overlapped. We predicted that functionally and spatially dissociated neuronal populations govern the representation of the three codes featured in the Triple Code Model [4]. Specifically, we predicted that visual Arabic representations of number (e.g. “3”) give rise to increased BOLD (Blood-Oxygen-Level Dependent) signal levels in the visual number form area (VNFA) in the right ITG. In line with the results of Schmithorst and Douglas Brown [35], Dehaene et al. [24], and the TCM, we furthermore expected to find that verbal representations of numerical quantity (e.g. “three”) give rise to increased BOLD signal levels in the left perisylvian language areas (primarily SMG, IFG, MTG, STG). We predicted that analog nonsymbolic magnitude representations (e.g. •••) give rise to increased BOLD signal levels in the bilateral horizontal segment of the intraparietal sulcus (hIPS; for reviews see [24, 52]). Finally, we predicted that the IPS would show consistent activation across all tasks, given its roles in semantic interpretation of symbolic numerical formats and comparative numerical operations [4, 24]. Atlas-based Region of Interest (ROI) analyses were performed for each of the hypothesized areas of interest, based on probability maps in the SPM Anatomy Toolbox [53] where applicable.

## Materials and methods

### Participants

Fifty-four right-handed, healthy adult students (ages 18-33) were recruited from Linköping University. None of the recruited participants had evidence or history of drug abuse or neurological illness. All participants had normal or corrected vision and normal color vision. None of the participants had any documented or self-reported mathematical difficulties and performed above chance on behavioral mathematical tasks, such as arithmetic fluency (i.e. the fast and accurate solving of arithmetic problems). Written informed consent was obtained from each participant, in line with the conditions for study approval presented by the Swedish Central Ethical Review Board (as approved by the Regional Ethical Review Board in Linköping, Sweden; study approval number: 2017/103-31). Following motion correction and realignment, six motion regressors were included in the first-level design matrix for each individual. Eight participants were excluded from further analyses due to excessive head motion (movement greater than 4 mm along any axis). The final sample consisted of 46 individuals (20 males and 26 females; *Mean age* = 23.83, *SD* = 3.28).

### Design and experimental tasks

**Fig 1:**
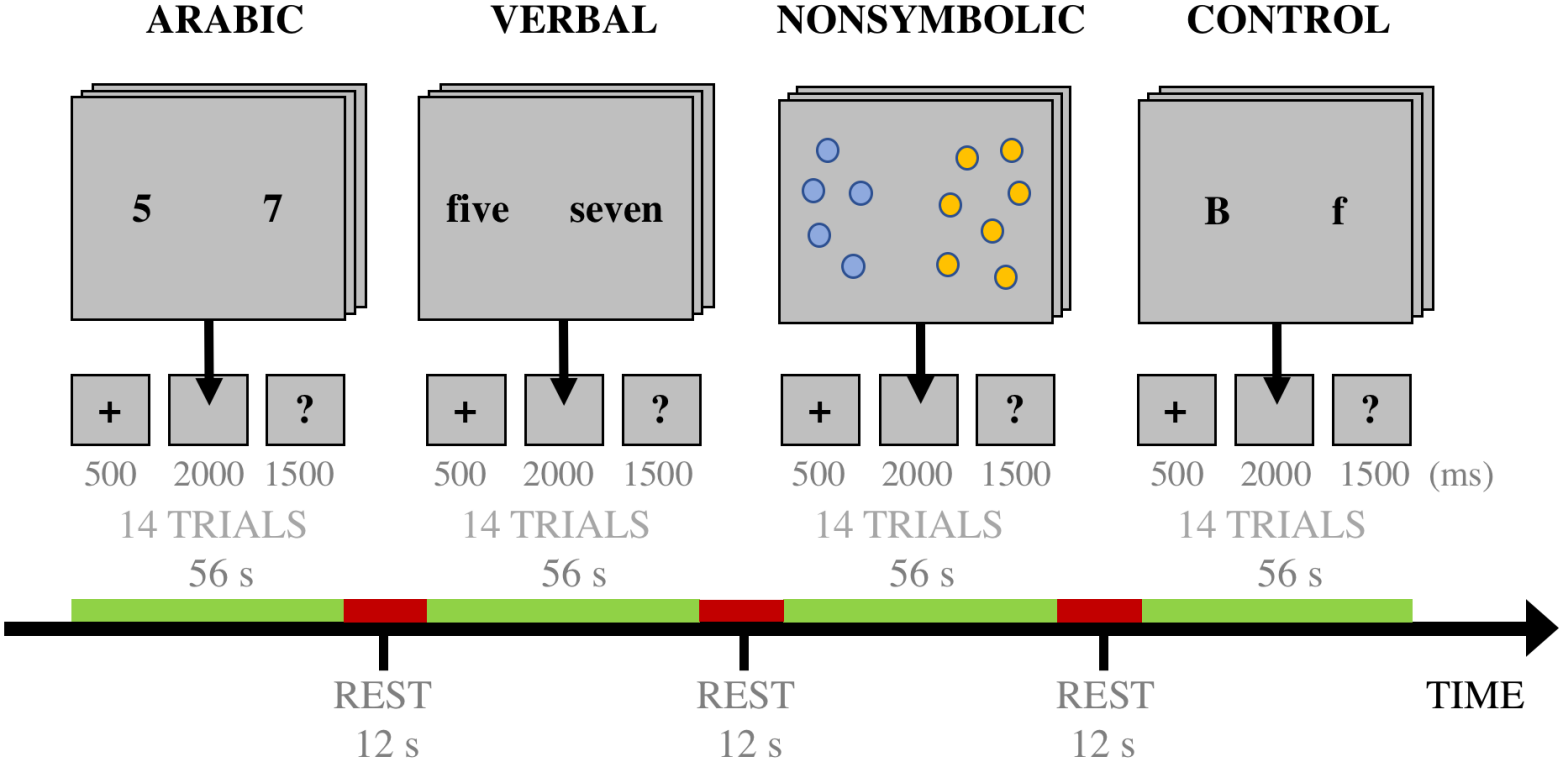
Overview of the experimental paradigm. Three experimental tasks and one control task were featured. One cycle of blocks is illustrated. Six such cycles were administered.

Three experimental tasks, one control task, and two unrelated tasks were administered sequentially over six experimental blocks, repeated for a total of six cycles. An alternating blocked design with a fixed task order was used in order to maximize contrast frequency (i.e. minimize time between similar tasks), due to the large number of included tasks, as recommended by Henson [54]. Distant and close number pairs were alternated in every other cycle of blocks (see Fig 1 for an overview of the relevant tasks included in the fMRI paradigm). A 12 second resting period was administered at the end of each block, in order to return the hemodynamic response signal to baseline levels. All tasks featured 14 trials per block, each of which was presented for a total of 4000 ms (including fixation cue, stimulus presentation, and response window) and resulted in a block duration of 56 seconds. Each block was preceded by a brief (2 seconds) instruction screen, indicating the start and purpose of the following task. The MRI protocol lasted for a total of 55 minutes. SuperLab 5 (Cedrus Corporation, San Pedro, CA, USA) was used to administer all tasks, and was used to record reaction time and accuracy data for each participant. Task responses were collected using a Lumina response pad (Cedrus Corporation, San Pedro, CA, USA). Participants were instructed to use buttons beneath their right-hand index and middle fingers to indicate their response, corresponding to the left and right-lateralized response options on the screen. All stimulus materials were presented visually, using a pair of VisuaStimDigital video goggles (Resonance Technology Inc., Northridge, CA, USA) placed directly over the eyes of each participant. Corrective lenses were used when necessary.

Each cycle of blocks was initiated by the Arabic digit comparison task, followed by the verbal number comparison task, the nonsymbolic magnitude comparison task, and ended with the control task. Parametric task difficulty levels were alternated for each such cycle. Two additional tasks, focused on the subjective judgment of risk associated with various activities, followed the control task. We do not expect that these tasks, given their similarities to experimental tasks in both stimulus presentation and response requirements (i.e. the consistent choice of a “larger” alternative, whether in terms of higher cardinal values or more dangerous activities), would interfere with the results of the experiment-relevant tasks. These tasks were unrelated to the current study and will be presented in detail elsewhere. Two longer periods of rest were placed after block cycles 2 and 4, spanning approximately six and ten minutes respectively. The first period of rest featured the collection of Diffusion Tensor Imaging (DTI) data, whereas the second period of rest featured the collection of resting-state fMRI data. We do not expect these resting periods to significantly influence the results of the experimental tasks, as participants were provided with an opportunity to recover and regain their concentration after completing several experimental trials. As for the additional tasks, these data will be presented in detail elsewhere.

Previous research has indicated that the difficulty of numerical discrimination increases in line with the distance effect, which increases response times and decreases response accuracies for closely spaced numbers (e.g. 5 vs 6), especially for numerically large number pairs (e.g. 8 vs 9) [15]. This effect has also been shown to apply during discrimination of nonsymbolic magnitudes, where a smaller ratio between two sets of objects (e.g. 3:4) results in a more taxing discrimination task than for larger ratios (e.g. 1:2) [55]. In order to control for task difficulty, we established a parametric design where all experimental tasks featured numerically “close” (e.g. 5 vs 6) and “distant” (e.g. 3 vs 7) stimulus pairs, corresponding to “difficult” and “easy” conditions for each task. Statistical [Close > Distant] contrasts were established for each experimental task, with the purpose of evaluating potential difficulty-related differences in the engagement of number-relevant brain areas.

#### Arabic digit comparison

The Arabic digit comparison task featured horizontally presented pairs of Arabic single digits, and required participants to select the numerically larger digit by pressing a corresponding button on the response pad. Over the course of one experimental block, participants were required to make 14 such judgements. Each trial was preceded by a fixation cross, displayed for 500 ms, after which a pair of Arabic digits were presented for 2000 ms. A response screen followed for 1500 ms, where participants received no feedback regarding their response. Two numerical distances were used in order to account for distant (4–5; e.g. 3 versus 7) and close (1–2; e.g. 4 versus 5) trials, which were alternated for each block.

#### Verbal number comparison

The verbal number comparison task featured horizontally presented pairs of verbal number words, each denoting a quantity in the single digit range (e.g. “three” versus “seven”). Participants were required to select the numerically larger number word, indicated by pressing a corresponding button on the response pad, for each of the 14 trials within one experimental block. A fixation cross was presented for 500 ms, followed by a pair of verbal number words presented for 2000 ms. Each trial ended with a response screen of 1500 ms, where participants received no feedback regarding their response. Two numerical distances were used to represent differences in task difficulty: 4–5 (distant trials; e.g. “three” versus “seven”) and 1–2 (close trials; e.g. “five” versus “six”). Task difficulty was alternated for each block.

#### Nonsymbolic magnitude comparison

The nonsymbolic magnitude comparison task was adapted from Halberda et al. [13]. Participants were required to discriminate between two simultaneously presented, horizontally separated (i.e. non-overlapping) arrays of blue and yellow dots (8–26 dots in total). For each of the 14 trials in a block, participants were instructed to select the most numerous array of dots by pressing the corresponding left or right-lateralized button on the response pad. In order to control for the effects of surface area in numerosity estimation, half of all trials featured a cumulative surface area congruent with the number of dots (i.e. cumulative surface area and number corresponded). The effects of cumulative surface area on numerosity estimation are debated [56], although recent experimental evidence increasingly suggests that object numerosity constitutes a more salient perceptual cue than cumulative surface area [57]. In this vein, DeWind and colleagues [34] modeled performance on nonsymbolic magnitude comparison tasks by modulating the size, spacing, and numerosity of dot arrays as orthogonal dimensions, in order to individually manipulate each of these dimensions and measure their effects on participants’ numerical choice behavior. These features are argued to be mutually dependent, as changes in numerosity between two sets of dots in an array necessarily determine both the distance between the centers of individual dots (as dependent on dot size) and the distance between sets of dots (as dependent on the spacing of a specific numerosity presented in a fixed size). However, in order to investigate whether numerosity itself was the driving factor behind numerical discrimination, these three dimensions were manipulated throughout the available parameter space in order to tease apart the influence of each such dimension. While non-numerical stimulus features were observed to influence numerosity discrimination performance, a majority of participants relied on numerosity as the primary visual cue in their choice behavior.

Each trial began with a fixation cross of 500 ms, followed by two stimulus dot arrays presented for 2000 ms. Each trial ended with a response screen of 1500 ms, where participants received no feedback regarding their response. Two numerosity ratios were used in order to represent differences in task difficulty: 1:2 (distant trials; e.g. 12 versus 24 dots) and 4:3 (close trials; e.g. 10 versus 13 dots).

#### Control task

A letter case discrimination task was used as a baseline control task for all experimental conditions, featured at the end of each cycle of blocks. Two letters were presented horizontally, where one was printed in uppercase and the other printed in lowercase (e.g. “A” versus “f”). Participants were requested to select the uppercase letter in each of the 14 trials in a block, meaning that the control task followed the same presentation format and response requirements as the experimental tasks. A fixation cross was presented for 500 ms at the onset of each trial, followed by a stimulus presentation of 2000 ms. Each trial ended with a response screen lasting 1500 ms. Participants responded by pressing the corresponding right or left-lateralized button on the response pad, and received no feedback regarding their response. Note that the task is superficially similar to previous experimental tasks, as the language of instruction, Swedish, colloquially refers to uppercase letters as “large letters” and to lowercase letters as “small letters”. We did not expect interfering patterns of activity for the control and Arabic digit comparison tasks, as previous research indicates the existence of dissociated functional regions for single letter (*Visual Word Form Area*) and single number (Visual Number Form Area) processing within the left fusiform gyrus [40, 43, 45, 58, 59]. The Visual Word Form Area has consistently been observed to invariantly encode lower- and uppercase single letters, with differences in activation present only for letter strings and words [59–61].

### fMRI data acquisition

The fMRI experiment was conducted at the Center for Medical Imaging and Visualization (CMIV), Linköping University. A 3.0 T Siemens Magnetom Prisma MRI scanner, fitted with a twenty-channel head coil, was used for data acquisition. Forty-eight 3.0 x 3.0 x 3.0 mm thick slices with an in-plane resolution of 3.0 mm isotropic and no gap were used. High-resolution structural scans were acquired using a T1-weighted pulse sequence (TR = 2300.0 ms, TE = 2.36 ms, flip = 8°, slice thickness = 0.90 x 0.90 x 0.90 mm, number of slices = 208) for each participant prior to the acquisition of functional scans, in order to assist localization and co-registration during data pre-processing. A BOLD-sensitive (Blood Oxygen Level Dependent) T2*-weighted ascending Echo Planar Imaging (EPI) pulse sequence was used to acquire whole-brain functional scans (TR = 1340.0 ms, TE = 30.0 ms, flip = 69°).

### fMRI data analysis

Pre-processing and general linear model (GLM) analysis was performed using SPM8 (Wellcome Department of Cognitive Neurology, London, UK). Functional images were motion corrected by realignment and by reference to the mean image. Co-registration was performed with the segmented anatomical image. Image smoothing was performed using a Gaussian kernel of 8 x 8 x 8 mm full width at half maximum (FWHM), and normalized using the default gray matter probability template in MNI (Montreal Neurological Institute) space. We initially planned to use a set significance threshold of *p* = .05 with familywise error-correction (FWE) for all contrasts. However, due to the statistical power attained by our participant sample, we chose a FWE-threshold of *p* = .01 in order to increase spatial specificity where possible. In cases where suprathreshold cluster activity was not found at the *p* = .01 or.05 FWE-corrected levels, a threshold of *p* = .001 uncorrected was used. For these analyses, the results should be viewed with caution and remain to be verified in future studies.

A whole-brain voxel-wise BOLD-analysis was performed for each subject and task, where each experimental condition was contrasted with the control condition and the corresponding [Close > Distant] (i.e. parametric comparison) task contrast. A second-level random effects analysis with a FWE threshold of *p* = .01 was performed in order to report group-level results. The degree of overlapping BOLD activity patterns for all experimental tasks was evaluated with a conjunction (null) analysis for both the task–control contrasts and the [Close > Distant] contrasts, performed using an FWE threshold of *p* = .05. Individual parametric contrast analyses were performed with a significance threshold of *p* = .001 uncorrected. Atlas-based ROI analyses were performed at the voxel-level FWE threshold of *p* < 0.05 for all areas included in our hypotheses (i.e. IPS, right ITG, left SMG, IFG, MTG, and STG), using probability maps featured in SPM Anatomy Toolbox [53]. In cases where atlas-based regions of interest were not available, we selected seed regions based on previous research (individual summaries for each ROI analysis are available in the Results section).

It should be noted that single-voxel activation clusters have been included as meaningful activity patterns in the description of the results. Such results are generally an uncommon occurrence when FWE thresholds are applied, especially to second-level random effects analyses at the group level, as this method of correction is highly conservative and has been argued to promote the inflation of false-negative (i.e. type II) results in fMRI research [62]. This is especially true at a corrected threshold of *p* < .01 FWE (although *p* < .05 FWE is by no means a liberal threshold), which would be even more so conservative if paired with a minimum cluster size threshold of, for instance, 20 voxels. More to the point, it could be argued that the lack of cluster size thresholding in this study promotes some degree of false-positive (i.e. type I) results, but this decision has been made in order to not bias the results towards large effects at the expense of smaller—potentially interesting—patterns of activity. Consider, for instance, the small spatial extent of anatomical subdivision hIP3 in the intraparietal sulcus, comprising an approximate 0.1102% (*SD* = ± 0.0675%) of total brain volume [63]. Specifically localized effects in this region should thus necessarily be small in spatial extent and undistinguishable from surrounding noise when subjected to both FWE and cluster size thresholding, which strongly motivates the decision to use a single correction method. As a final note, the risk of false-positive results is greatly mitigated by the fact that such results do not tend to survive meta-analyses and replication attempts, whereas the omission of false-negative results prevents a principled examination of potentially important neural substrates; regardless of their spatial extent (see [62]).

## Results

### Behavioral results

Behavioral data was collected for 44 out of the 46 included participants in this study. The reaction times (RTs) and accuracies for each experimental task were analyzed using two separate Bonferroni-corrected repeated-measures Analyses of Variance (ANOVA), and evaluated post-hoc with Bonferroni-corrected paired-samples t-tests. A summary of behavioral results can be found in Table 1 and S1 Fig.

**Table 1:** Overview of behavioral results.

Condition	Reaction time (ms)		Accuracy	
	*M*	*SD*	%	*SD*
Arabic (distant trials)	483.15	76.18	94.64	4.69
Arabic (close trials)	460.31	82.05	93.93	4.72
Verbal (distant trials)	483.39	82.70	95.78	4.26
Verbal (close trials)	469.22	85.18	93.24	4.15
Nonsymbolic (distant trials)	490.44	92.12	95.02	4.17
Nonsymbolic (close trials)	500.37	86.53	90.35	3.99
Control condition	472.73	82.49	94.18	4.01

Reaction time and response accuracy data from 44 (out of 46) participants.

A significant difference in reaction times across tasks was found, *F*(3.93, 169.18) = 9.99, *p* < 0.001, *η*^2^_p_ = 0.19. Nine paired-samples t-tests were performed for each task–control and parametric contrast in cross-condition comparisons, in order to investigate these effects. Following Bonferroni correction, significant differences in reaction times were found for the parametric comparisons of the Arabic task, *t*(43) = 3.94, *p* < 0.001; the verbal task, *t*(43) = 2.58, *p* < 0.001; and the nonsymbolic task, *t*(43) = -1.50, *p* = 0.01. Cross-condition comparisons revealed significant differences in reaction times between the [Distant] Arabic and nonsymbolic tasks, *t*(43) = -1.02, *p* = 0.03; the [Distant] verbal and nonsymbolic tasks, *t*(43) = -0.91, *p* = 0.04; the [Close] Arabic and verbal tasks, *t*(43) = -1.90, *p* = 0.007; the [Close] Arabic and nonsymbolic tasks, *t*(43) = -6.48, *p* < 0.001; and the [Close] verbal and nonsymbolic tasks, *t*(43) = -5.01, *p* < 0.001. Six paired-samples t-tests were used in order to compare the reaction times of experimental tasks to the control task. Significant differences were found for the [Distant] Arabic trials, *t*(43) = 1.68, *p* = 0.02; the [Distant] verbal trials, *t*(43) = 1.85, *p* = 0.01; the [Distant] nonsymbolic trials, *t*(43) = 2.46, *p* = 0.003; the [Close] Arabic trials, *t*(43) = -2.45, *p* = 0.003; and the [Close] nonsymbolic trials, *t*(43) = 3.47, *p* < 0.001.

A repeated-measures ANOVA also illustrated a significant difference in response accuracy across tasks, *F*(3.61, 155.09) = 10.88, *p* < 0.001, *η*^2^_p_ = 0.20. Post-hoc analysis was performed using nine paired-samples t-tests for each task–control and parametric contrast. Following Bonferroni correction, significant differences in response accuracy were found for the parametric comparisons of the Arabic task, *t*(43) = 0.70, *p* = 0.05; the verbal task, *t*(43) = 4.37, *p* < 0.001; and the nonsymbolic task, *t*(43) = 6.30, *p* < 0.001. Cross-condition comparisons revealed significant differences in reaction times between the [Distant] Arabic and verbal tasks, *t*(43) = -1.58, *p* = 0.01; [Distant] verbal and nonsymbolic tasks, *t*(43) = 1.31, *p* = 0.02; [Close] Arabic and verbal tasks, *t*(43) = 0.86, *p* = 0.04; [Close] Arabic and nonsymbolic tasks, *t*(43) = 3.09, *p* < 0.001; and the [Close] verbal and nonsymbolic tasks, *t*(43) = 3.55, *p* < 0.001. Six paired samples t-tests were used in order to compare the response accuracies of experimental tasks to the control task. Significant differences were found for the [Distant] verbal trials, *t*(43) = 3.26, *p* < 0.001; the [Distant] nonsymbolic trials, *t*(43) = 1.61, *p* = 0.02; the [Close] verbal trials, *t*(43) = -2.10, *p* = 0.007; and the [Close] nonsymbolic trials, *t*(43) = -5.15, *p* < 0.001.

### Brain imaging results

The SPM Anatomy Toolbox [53] was used to report probabilistic cytoarchitectonic maps of task-related activation for all featured contrasts.

#### Arabic digit comparison

**Fig 2:**
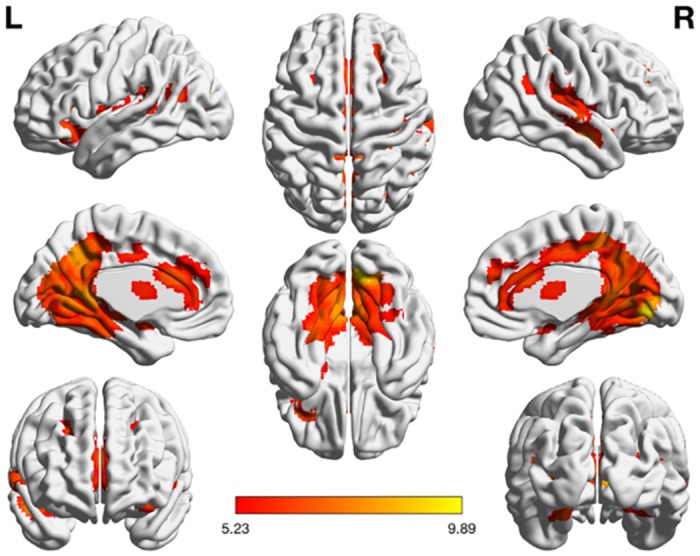
Activation maps of the Arabic digit comparison task. The task was contrasted with the control condition (*p* < 0.01 FWE).

The Arabic digit comparison analyses were designed to determine task-related activation for Arabic numbers. All relevant Arabic tasks were contrasted with the baseline control condition. The activation patterns related to the Arabic digit comparison task (see Table 2 and Fig 2) were primarily elicited in the occipital and temporal lobes, insula, anterior cingulate cortex, superior frontal gyrus, amygdala, and right angular gyrus (IPS).

**Table 2:** Clusters identified in the Arabic > control contrast (FWE-corrected *p* < 0.01).

Anatomical region	MNI coordinates	Cluster size	*p*	*Z* -score
Right Lingual Gyrus (hOc2)	9, –79, –4	3056	< 0.001	7.17
Left Middle Occipital Gyrus	–21, –82, 14		< 0.001	6.42
Left Precuneus	–6, –55, 38		< 0.001	6.40
Right Middle Temporal Gyrus	48, –13, –19	623	< 0.001	6.21
Right Insula Lobe (Ig2)	39, –19, –4		< 0.001	5.81
Right Superior Temporal Gyrus (TE 1.0)	57, –19, 5		< 0.001	5.54
Left Anterior Cingulate Cortex	–3, 23, 20	220	< 0.001	5.66
Left Anterior Cingulate Cortex	0, 41, 5		0.001	5.26
Left Medial Frontal Gyrus	3, 47, 23		0.001	5.21
Left Inferior Frontal Gyrus (p. Orbitalis)	–36, 29, –10	57	< 0.001	5.66
Left Inferior Frontal Gyrus (p. Triangularis)	–42, 29, 5		0.002	5.02
Left Superior Temporal Gyrus	–51, –37, 11	50	< 0.001	5.43
Left Caudate Nucleus	–3, –1, 11	44	0.002	5.03
Right Caudate Nucleus	6, 2, 11		0.006	4.73
Right Caudate Nucleus	9, 11, 8		0.008	4.67
Left Insula Lobe (Ig2)	–42, –16, 2	35	< 0.001	5.30
Left Superior Temporal Gyrus (OP4 [PV])	–54, –4, 5		0.003	4.91
Right Angular Gyrus (PGa/hIP1)	48, –52, 29	21	0.002	4.96
Right Superior Frontal Gyrus	21, 32, 38	19	< 0.001	5.38
Right Superior Frontal Gyrus	21, 23, 44		< 0.001	5.30
Right Superior Frontal Gyrus	21, 41, 32		0.001	5.27
Left Amygdala (CM)	–24, –1, –16	18	0.002	5.02
Left Insula Lobe	–27, 11, –13		0.004	4.80
Right Caudate Nucleus	18, 20, 2	3	0.006	4.74
Right Caudate Nucleus	15, –7, 23	2	0.005	4.78
Left Middle Frontal Gyrus	–24, 14, 44	1	0.005	4.77
Left Middle Frontal Gyrus	–27, 17, 41	1	0.006	4.74
Left Insula Lobe	–39, 11, –13	1	0.007	4.68
Left Medial Frontal Gyrus	–12, 35, 35	1	0.009	4.64
Left Insula Lobe (Ig1)	–33, –19, 14	1	0.009	4.62

Coordinates indicate peak-level activation. Cluster size indicates number of voxels.

**Fig 3:**
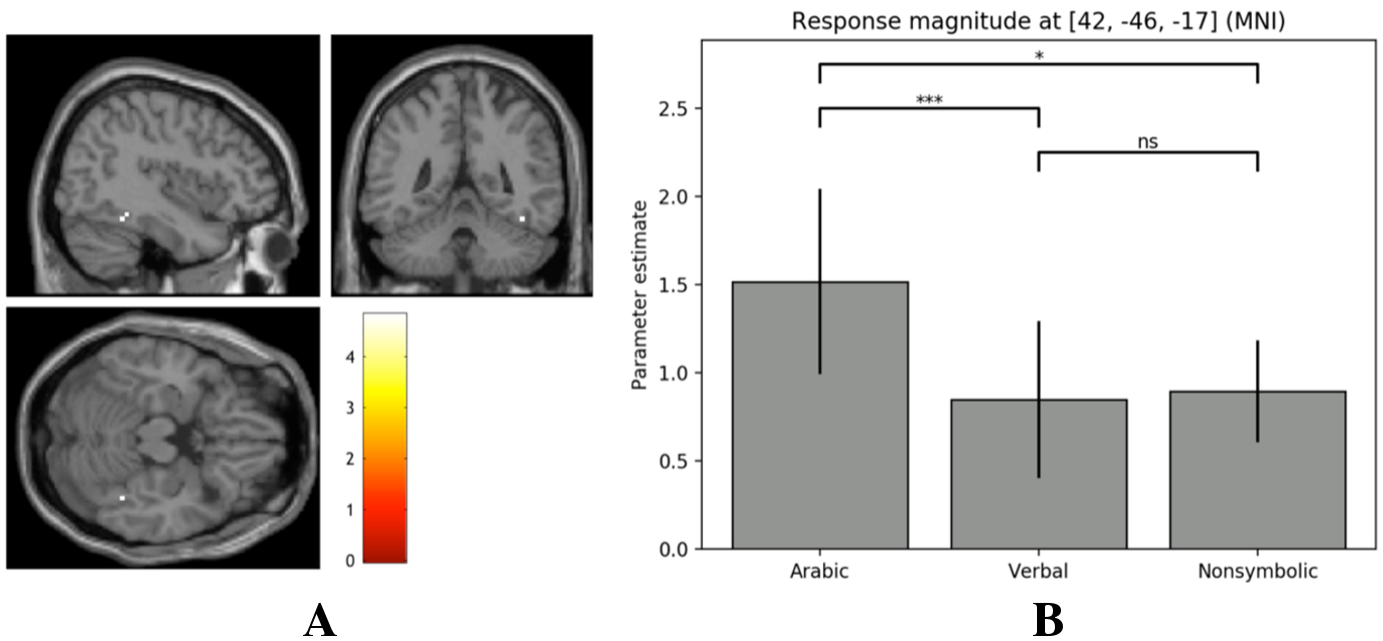
VNFA cluster activity and response magnitudes. (A) Slices illustrate VNFA cluster activity at z = -17 across conditions (*p* < 0.05 FWE). (B) Average parameter estimates for this cluster across conditions.

A region of interest analysis was performed using a 15 mm spherical ROI, centered at MNI coordinates 56, -47, -18. These coordinates were chosen on the basis of being the mean of two frequently cited locations of the VNFA (55, -43, -20; 57, -51, -17) [42, 43, 64, 65]. We initially planned to use a sphere size of 5 mm, as in Park et al. [66], but chose to increase the size in order to reach a suprathreshold activity cluster. This decision was furthermore motivated by the largely inconsistent placement of the VNFA in the literature, with potential neural substrates located throughout the ITG and MTG [42]. Significant activity was found at MNI coordinates 42, -46, -17 (cluster size = 2 voxels, *Z* = 4.30, *p* = 0.033 at *p* < 0.05 FWE), located in the right ITG. Following the procedure of Park and colleagues [66], beta values were extracted from the ROI in order to compare response magnitudes across conditions, as a means of investigating whether the observed activity could be exclusively attributed to Arabic number processing. A repeated-measures ANOVA illustrated a significant difference in mean beta values across conditions, *F*(1.55, 69.77) = 3.69, *p* = 0.040, *η*^2^_p_ = 0.076. Three Bonferroni-corrected paired-samples t-tests were performed as a post-hoc analysis, revealing a significant difference in mean ROI beta values for the Arabic and verbal tasks, *t*(45) = 3.37, *p* < 0.001, as well as between the Arabic and nonsymbolic tasks, *t*(45) = 1.87, *p* = 0.02. These results suggest that the voxel cluster at 42, -46, -17 (MNI) preferentially responds to Arabic numerals, and may represent a possible location of the VNFA (see Fig 3).

An atlas-based ROI analysis was performed on the bilateral IPS, illustrating significant activity in area hIP1, located in the right anterior IPS (MNI: 42, -52, 32, cluster size = 1 voxel, *Z* = 4.18, *p* = 0.049 at *p* < 0.05 FWE).

#### Verbal number comparison

**Fig 4:**
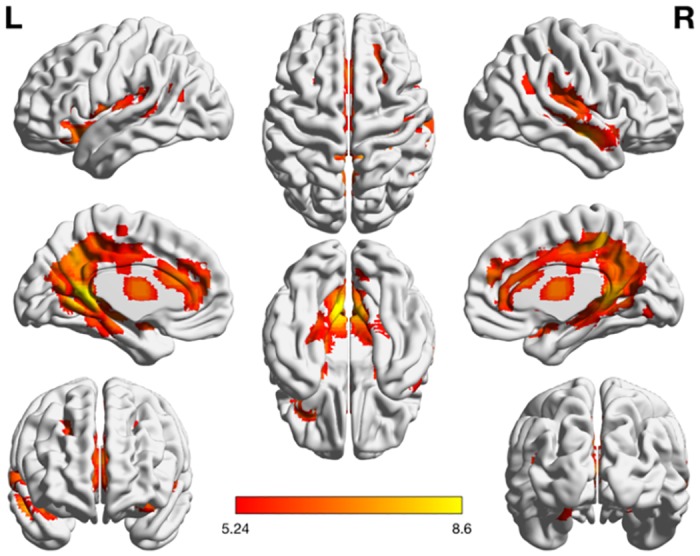
Activation maps of the verbal number comparison task. The task was contrasted with the control condition (*p* < 0.01 FWE).

The verbal number comparison analyses were designed to determine task-related activation, by contrasting all relevant verbal tasks to the baseline control condition. The activation patterns related to the verbal number comparison task (see Table 3 and Fig 4) were primarily elicited in the occipital and temporal lobes, precuneus, superior and inferior temporal gyri, anterior cingulate cortex, insula, thalamus, hippocampus, superior frontal gyrus, middle temporal gyrus, and right angular gyrus (IPS).

**Table 3:** Clusters identified in the verbal > control contrast (FWE-corrected *p* < 0.01).

Anatomical region	MNI coordinates	Cluster size	*p*	*Z* -score
Left Precuneus	–6, –52, 8	2023	< 0.001	6.58
Right Paracentral Lobule (5Ci)	15, –40, 50		< 0.001	6.09
Right Precuneus	9, –49, 8		< 0.001	6.06
Right Middle Temporal Gyrus	48, –13, –19	889	< 0.001	6.18
Right Posterior Insula (Ig2)	39, –19, –1		< 0.001	5.84
Right Superior Temporal Gyrus (OP1)	57, –19, 11		< 0.001	5.55
Left Inferior Frontal Gyrus (p. Orbitalis)	–33, 26, –13	317	< 0.001	5.64
Left Insula Lobe	–27, 11, –13		< 0.001	5.58
Left Insula Lobe (Ig2)	–42, –16, 2		< 0.001	5.52
Left Anterior Cingulate Cortex	–3, 26, 20	240	< 0.001	5.65
Right Medial Frontal Gyrus	3, 47, 23		< 0.001	5.49
Left Anterior Cingulate Cortex	0, 41, 8		< 0.001	5.48
Thalamus (Temporal)	–3, –4, 11	142	< 0.001	5.62
Thalamus (Temporal)	–9, –7, 20		< 0.001	5.58
Left Hippocampus	–24, –22, –13	129	< 0.001	5.53
Left ParaHippocampal Gyrus	–27, –37, –10		< 0.001	5.33
Right Caudate Nucleus	15, –7, 23	21	0.002	5.04
Right Caudate Nucleus	15, 5, 20		0.005	4.78
Right Angular Gyrus (PGa/hIP1)	48, –52, 29	11	0.003	4.93
Right Superior Frontal Gyrus	24, 20, 44	8	0.001	5.14
Right Caudate Nucleus	18, 17, 11	6	0.005	4.79
Right Caudate Nucleus	18, 20, 2		0.008	4.67
Left Middle Temporal Gyrus	–45, –58, 20	5	0.002	5.00
Right Superior Frontal Gyrus	21, 32, 38	3	0.001	5.28
Right Caudate Nucleus	9, 11, –1	3	0.004	4.80
Right Lingual Gyrus (hOc2)	12, –79, –4	3	0.006	4.72
Left Inferior Frontal Gyrus (p. Triangularis)	–42, 26, 5	2	0.004	4.83
Left Posterior-Medial Frontal Cortex (pMFC)	–6, –19, 59	2	0.007	4.70
Right Superior Frontal Gyrus	21, 41, 32	1	0.002	5.02
Right Precentral Gyrus	21, –16, 56	1	0.006	4.72
Right Insula Lobe	36, 8, 8	1	0.007	4.69
Right Caudate Nucleus	9, 11, 8	1	0.009	4.64
Left Primary Somatosensory Cortex (Area 3a)	–18, –31, 56	1	0.009	4.63
Left Middle Frontal Gyrus	–24, 14, 44	1	0.010	4.61

Coordinates indicate peak-level activation. Cluster size indicates number of voxels.

Four region of interest analyses were performed in line with our hypotheses, targeting the left inferior frontal gyrus, left superior temporal gyrus, left supramarginal gyrus, and left middle temporal gyrus. Atlas-based probability maps in SPM Anatomy Toolbox [53] were used for the IFG (Broca’s area; area 44 and 45), STG (Wernicke’s area; anatomical subdivision TE3 [67]), and SMG (anatomical subdivision PF, argued to be part of a dorso-dorsal semantic language processing network [68, 69]). Following the method in Park et al. [66], a spherical ROI measuring 5 mm was centered around the peak-level left MTG activation cluster identified in the [Verbal > Control] contrast.

Significant activity was found in the IFG, at MNI coordinates -51, 8, 2 (cluster size = 2 voxels, *Z* = 4.44, *p* = 0.019 at *p* < 0.05 FWE), located in the left rolandic operculum (area 44). Beta value response magnitudes were not compared across conditions for this area, as no suprathreshold clusters were found in the other conditions.

**Fig 5:**
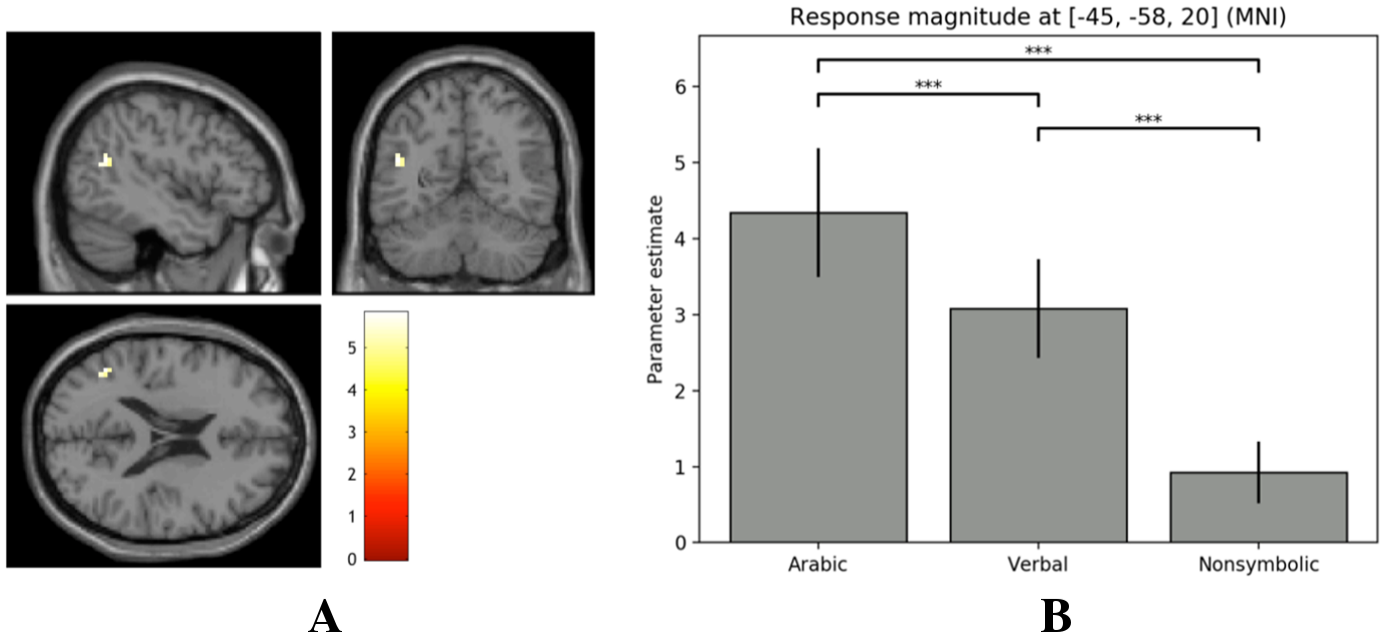
MTG cluster activity and response magnitudes. (A) Slices illustrate MTG cluster activity at z = 20 across conditions (*p* < 0.05 FWE). (B) Average parameter estimates for this cluster across conditions.

A region of interest analysis of the left MTG activation cluster (centered at MNI: -45, -58, 20) revealed a significant cluster of 10 voxels (*Z* = 5.00, *p* = 0.002 at *p* < 0.05 FWE). A repeated-measures ANOVA illustrated a significant difference in mean beta values across conditions, *F*(1.42, 63.99) = 30.30, *p* < 0.001, *η*^2^_p_ = 0.402. Three paired-samples t-tests were used to investigate this effect further, revealing that larger response magnitudes of this area were primarily associated with Arabic and verbal (i.e. symbolic) tasks. A significant difference was found between mean ROI beta values for the Arabic and verbal tasks, *t*(45) = 3.77, *p* < 0.001; the Arabic and nonsymbolic tasks, *t*(45) = 6.04, *p* < 0.001; and the verbal and nonsymbolic tasks, *t*(45) = 5.39, *p* < 0.001 (see Fig 5).

Region of interest analyses centered on area PF in the supramarginal gyrus as well as on Wernicke’s area (TE3) did not demonstrate suprathreshold activation.

An atlas-based ROI analysis was performed on the bilateral IPS, illustrating significant activity in area hIP1, located in the right anterior IPS (MNI: 42, -52, 32, cluster size = 2 voxels, *Z* = 4.37, *p* = 0.025 at *p* < 0.05 FWE).

#### Nonsymbolic magnitude comparison

**Fig 6:**
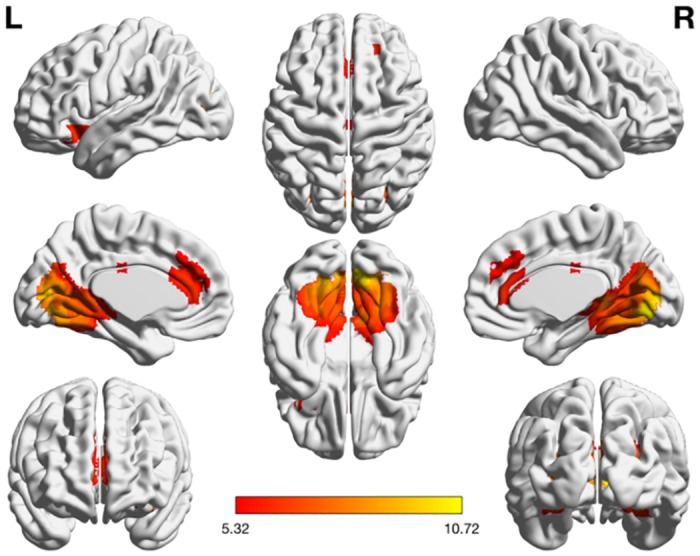
Activation maps of the nonsymbolic magnitude comparison task. The task was contrasted with the control condition (*p* < 0.01 FWE).

The nonsymbolic magnitude comparison analyses were designed to contrast all relevant nonsymbolic tasks to the baseline control condition. The activation patterns related to the nonsymbolic magnitude comparison task (see Table 4 and Fig 6) were primarily elicited in the occipital and frontal lobes, anterior cingulate cortex, middle cingulate cortex, inferior and superior frontal gyri.

**Table 4:** Clusters identified in the nonsymbolic > control contrast (FWE-corrected *p* < 0.01).

Anatomical region	MNI coordinates	Cluster size	*p*	*Z* -score
Right Lingual Gyrus (hOc2)	9, –76, –7	2386	< 0.001	7.51
Left Lingual Gyrus (hOc3v)	–15, –73, –4		< 0.001	7.29
Left Middle Occipital Gyrus	–21, –82, 14		< 0.001	7.25
Left Anterior Cingulate Cortex	–3, 32, 20	179	< 0.001	5.37
Left Anterior Cingulate Cortex	0, 41, 8		< 0.001	5.36
Left Middle Cingulate Cortex	0, 29, 32		0.004	4.86
Left Inferior Frontal Gyrus (p. Orbitalis)	–33, 20, –13	36	< 0.001	5.35
Right Superior Frontal Gyrus	21, 41, 32	2	0.001	5.11
Right Caudate Nucleus	9, 14, 2	2	0.007	4.76
Right Olfactory Cortex	27, 11, –13	1	0.006	4.77
Right Primary Somatosensory Cortex (Area 3b)	24, –37, 53	1	0.008	4.71
Right Middle Cingulate Cortex	0, –19, 29	1	0.010	4.67

Coordinates indicate peak-level activation. Cluster size indicates number of voxels.

A region of interest analysis centered on the bilateral IPS did not demonstrate significant suprathreshold activation.

#### Comparison of parametric contrasts

**Fig 7:**
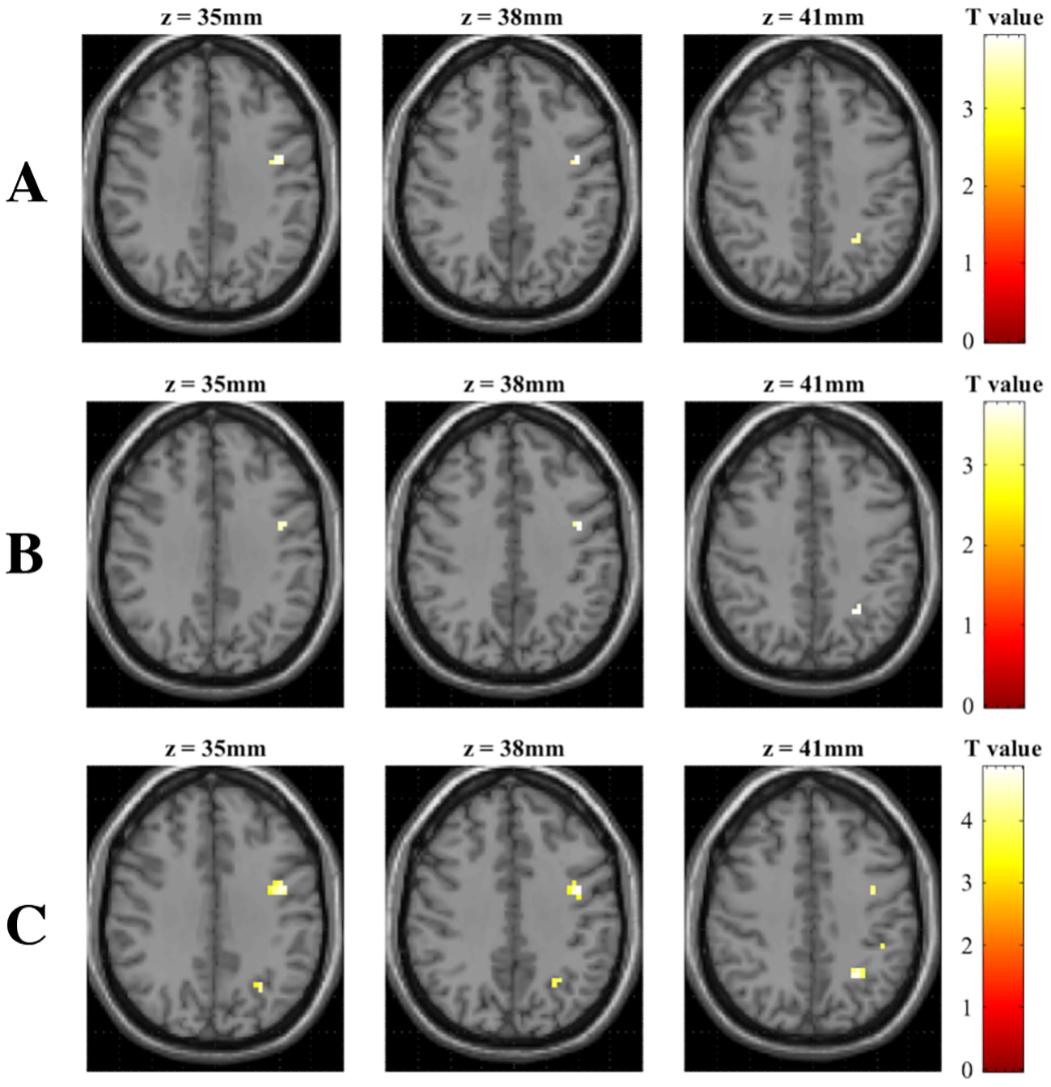
Activation maps of each parametric task contrast. All contrasts were thresholded at *p* < 0.001 uncorrected. Slices range from z = 35 to z = 41. (A) Arabic digit comparison task. (B) Verbal number comparison task. (C) Nonsymbolic magnitude comparison task.

The [Close > Distant] contrast of each experimental condition was analyzed to determine whether notable differences in numerical distance could be identified in task-related activation (see Table 5 and Fig 7). Activity in the left Inferior Parietal Lobule (area IPS) was found for all three experimental conditions. All [Close > Distant] contrasts also demonstrated activity in the left Precentral Gyrus, whereas the nonsymbolic [Close > Distant] contrast additionally featured activity in anatomical subdivision PFt of the left IPL [68].

**Table 5:** Clusters identified in the parametric [Close > Distant] contrasts (uncorrected *p* < 0.001).

Contrast	Anatomical region	MNI coordinates	Cluster size	*p*	*Z* -score
Arabic [Close > Distant]	Left Precentral Gyrus	–42, –1, 35	9	< 0.001	3.63
	Left Inferior Parietal Lobule (hIP3)	–27, –52, 44	7	< 0.001	3.94
Verbal [Close > Distant]	Left Precentral Gyrus	–39, –1, 38	6	< 0.001	3.49
	Left Inferior Parietal Lobule (hIP3)	–27, –52, 44	6	< 0.001	4.11
Nonsymbolic [Close > Distant]	Left Precentral Gyrus	–39, –4, 38	30	< 0.001	4.29
	Left Inferior Parietal Lobule (hIP3)	–27, –52, 44	18	< 0.001	5.07
	Left Middle Occipital Gyrus (hIP3)	–27, –61, 35		< 0.001	3.85
	Left Inferior Parietal Lobule (PFt)	–42, –37, 41	1	< 0.001	3.48

Coordinates indicate peak-level activation. Cluster size indicates number of voxels.

#### Conjunction analysis of numerosity processing

**Fig 8:**
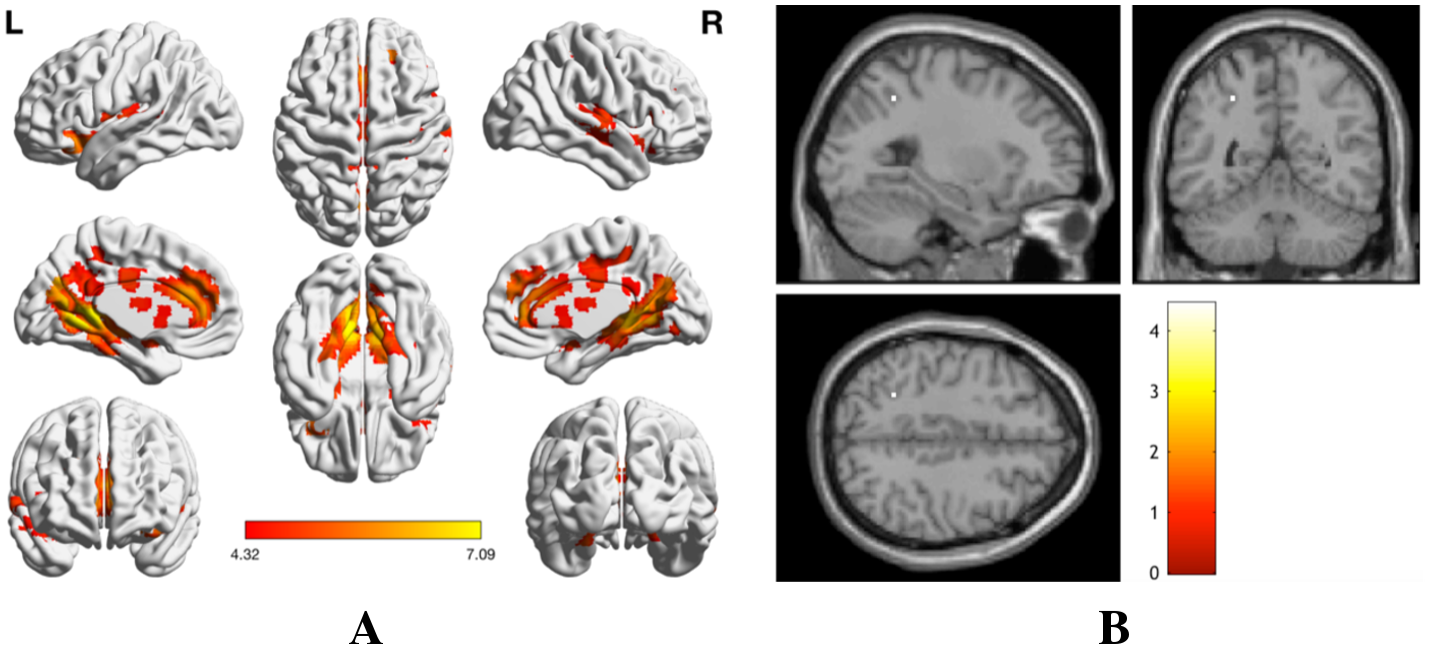
Overlapping activations across all tasks. (A) Conjunction analysis of all task–control contrasts (*p* < 0.05 FWE). (B) Conjunction analysis of parametric contrasts across all three tasks (*p* < 0.05 FWE).

A conjunction (null) analysis over the three task–control contrasts, [Arabic > Control] ∩ [Verbal > Control] ∩ [Nonsymbolic > Control], was performed in order to infer overlapping activation patterns and pathways across the components of the Triple Code Model (see Table 6 and Fig 8). Activation patterns were found in the occipital lobe, anterior, middle and posterior cingulate cortices, inferior and superior frontal gyri, superior temporal gyrus, insula, amygdala, thalamus, and caudate nucleus. The conjunction (null) analysis over the three parametric contrasts (i.e. [Close > Distant]; see Table 7) revealed consistent activation of the left Inferior Parietal Lobule (area IPS).

**Table 6:** Overlapping clusters identified in the conjunction task–control analysis (FWE-corrected *p* < 0.05).

Anatomical region	MNI coordinates	Cluster size	*p*	*Z* -score
Left Lingual Gyrus (hOc2)	–9, –49, 2	1497	< 0.001	6.53
Left Calcarine Gyrus (hOc1)	–6, –61, 8		< 0.001	6.22
Left Calcarine Gyrus (hOc3d)	–9, –70, 23		< 0.001	6.08
Left Anterior Cingulate Cortex	–3, 26, 20	398	< 0.001	5.73
Left Anterior Cingulate Cortex	0, 41, 8		< 0.001	5.68
Right Anterior Cingulate Cortex	3, 47, 20		< 0.001	5.27
Left Inferior Frontal Gyrus (p. Orbitalis)	–33, 26, –13	212	< 0.001	5.61
Left Insula Lobe	–30, 14, –13		0.001	5.19
Left Insula Lobe	–39, 11, –13		0.001	5.18
Right Primary Somatosensory Cortex (Area 3b)	24, –37, 53	183	0.002	5.02
Left Middle Cingulate Cortex	–3, –19, 32		0.005	4.78
Right Middle Cingulate Cortex	9, –13, 41		0.007	4.67
Right Amygdala	27, 5, –16	181	0.003	4.85
Right Caudate Nucleus	9, 11, 8		0.004	4.78
Right Caudate Nucleus	15, 17, 2		0.005	4.75
Right Dysgranular Insula (Id1)	36, –19, –4	150	0.002	5.01
Right Granular Insula (Ig2)	39, –13, 5		0.004	4.80
Right Hippocampus	42, –22, –13		0.005	4.74
Right Superior Temporal Gyrus (TE 1.0)	54, –13, 2	102	0.004	4.81
Right Superior Temporal Gyrus (TE 3)	63, –16, 2		0.005	4.78
Right Superior Temporal Gyrus	57, –28, 8		0.008	4.65
Thalamus (Temporal)	3, –10, 14	38	0.015	4.49
Left Caudate Nucleus	–9, 5, 11		0.028	4.34
Left Caudate Nucleus	–12, –1, 17		0.029	4.33
Left Superior Parietal Lobule (5Ci)	–15, –46, 47	21	0.009	4.62
Left Middle Cingulate Cortex (5Ci)	–15, –37, 41		0.022	4.39
Right Inferior Frontal Gyrus (p. Triangularis)	48, 26, 5	7	0.017	4.46
Right Posterior Cingulate Cortex	9, –43, 26	5	0.013	4.52
Right Superior Frontal Gyrus	21, 41, 32	4	< 0.001	5.38
Right Lingual Gyrus (hOc2)	9, –79, –4	4	0.010	4.59
Right Temporal Pole (TE 3)	54, 14, –7	4	0.024	4.38
Left Caudate Nucleus	–9, 14, 8	2	0.033	4.29
Right Caudate Nucleus	15, –10, 20	2	0.038	4.25
Right Inferior Frontal Gyrus (p. Orbitalis)	39, 23, –16	1	0.032	4.30

Coordinates indicate peak-level activation. Cluster size indicates number of voxels.

**Table 7:** Overlapping clusters identified in the conjunction [Close > Distant] analysis (FWE-corrected *p* < 0.05).

Anatomical region	MNI coordinates	Cluster size	*p*	*Z* -score
Left Inferior Parietal Lobule (hIP3)	–27, –52, 44	1	< 0.001	4.29

Coordinates indicate peak-level activation. Cluster size indicates number of voxels.

## Discussion

The purpose of this study was to investigate the neural correlates of the Triple Code Model [4] within one experimental fMRI-paradigm, featuring tasks that appropriately target all three numerical codes: Arabic digit comparison, verbal number word comparison, and nonsymbolic magnitude comparison. This approach to the study of numerical cognition was designed to bridge an important gap in the current literature, as the Triple Code Model is a leading but partially explored theoretical foundation of the field [8]. We hypothesized that each numerical code would exhibit task-specific activation patterns, in line with independent findings of previous research. We expected to find activation in the right ITG (Visual Number Form Area) during Arabic digit comparison tasks (for a review, see Yeo et al. [42]). A cluster in the right ITG indeed demonstrated activity during the Arabic digit comparison task, and was furthermore observed to preferentially respond to Arabic digits in comparison with verbal and nonsymbolic stimuli. However, this pattern of activity was not observed as peak-level activity in the whole-brain analysis of Arabic digit comparison tasks, but rather as part of a larger cluster (623 voxels) which demonstrated peak activity in the right middle temporal gyrus. For verbal number word comparison tasks, we expected the key involvement of left perisylvian language areas (SMG, IFG, MTG, and STG) [24, 35]. While task-related activity was elicited in the IFG and MTG, we did not observe suprathreshold activations in the left SMG or STG. The IPS was expected to be of primary importance for the nonsymbolic magnitude comparison tasks [24, 52, 70], but also active during all other numerical comparison tasks due to its special status as primary neural correlate of numerical cognition [5, 20, 21]. The observed patterns of IPS activity were surprising, as only the right anterior subdivision hIP1 was found to be active during symbolic number comparison tasks, whereas no suprathreshold activity was found for the nonsymbolic magnitude comparison task. However, the parametric comparison of task difficulty revealed activity in the left hIP3 for the [Close > Distant] contrasts in all experimental conditions. We furthermore expected that a conjunction analysis of all task-related activity would reveal the existence of a fronto-parietal network for numerical cognition, primarily implicating areas IPS, SPL, SFG, MFG, SMA, and ACC [44, 50]. Areas SPL, SFG, and ACC were found to be elicited in this analysis, while the SMA, IPS, and MFG did not demonstrate suprathreshold activity.

In the context of the TCM, the observed activity highlights a need to account for bi-hemispheric involvement in all numerical codes. While the purpose of this study was to evaluate the neural underpinnings of numerical cognition as expressed by this model, the results indicate that Dehaene and Cohen’s [71] functional–anatomical account of the TCM appears coarse and partially incompatible with recent evidence. Whereas our hypotheses align with the original model, the results indicate an approximately even distribution of right and left-lateralized functional areas across all tasks. Recent research geared towards functional and structural connectivity analyses increasingly highlights the importance of bi-hemispheric and contralateral connectivity for numerical competence [8, 72], suggesting that the analysis of individual functional regions provides an incomplete account of numerical cognition. As will be debated over the following sections, the current results indicate a need to minimally include functional networks associated with visuospatial attention in the TCM, in order to align the model with contemporary empirical evidence. Arsalidou and Taylor [22] have proposed a list of recommended updates to the TCM, featuring several regions found in the current study. These functional regions include the right superior frontal gyrus, left inferior frontal gyrus, insula (which was absent in the [Nonsymbolic > Control] contrast), left anterior cingulate cortex, and right angular gyrus (which overlapped with the right hIP1 in the current study). These additions, corroborated by the current results, are interesting with respect to the salience network—tasked with inducing behavioral responses to salient external stimuli—and its functional association with the posterior parietal cortex (PPC; constituting the superior and inferior parietal lobule), ventromedial prefrontal cortex (VMPFC; a part of the default mode network), and dorsolateral prefrontal cortex (DLPFC; a part of the central-executive network) [73]. This interaction of functional regions provides a reason to argue that the fronto-parietal network of numerical cognition cooperates significantly with the salience, default mode, and central-executive networks, which should be investigated in future studies. Arsalidou and Taylor [22] furthermore suggested the inclusion of the precentral gyrus and cerebellum, which were not identified in the task–control contrasts. In light of the need to revise the TCM, we propose the additional inclusion of primary somatosensory area 3b and visual areas V2 and V3.

### Arabic digit comparison

Task-specific activation for the Arabic digit comparison task was contrasted with the control condition (at a threshold of *p* < .01 FWE), revealing a pattern of activation extending throughout the ventral and dorsal streams (for a review of the two-streams hypothesis, see e.g. [74]). Notably, significant activation was found in early (right hOc2) V2 dorsal-steam visual areas. Nonsymbolic magnitude processing has recently been argued to begin in the early visual stream following findings of numerosity-sensitive retinotopic maps in areas V2 and V3 [30, 75], and the current results show robust similar patterns across all conditions. These results suggest that not only nonsymbolic magnitude representations are processed in early visual areas, but also symbolic numerosity; extending the results of Fornaciai et al. [30] and Roggeman et al. [31] to encompass the three codes of the Triple Code Model. While it could be the case that the visual presentation of stimuli primarily accounts for activity in early visual areas, we would expect the similar presentation formats found in the symbolic and control tasks to subtract activity related to non-numerical visual features. Given the novelty of these results, future research should investigate the involvement of early visual areas for all numerical representational formats further.

A region of interest analysis was performed in the right ITG (at a threshold of *p* < .05 FWE), which has previously been reported as a likely candidate area for the VNFA [42]. Although we deviated from the precedent established by Park et al. [66] with a three-fold increase in search space, we opted to provide explorative results in light of the previously inconsistent localization of the VNFA [36, 42]. Task-related activity for Arabic digits was found at MNI coordinates 42, -46, -17, located within the right ITG. Moreover, a comparison of parameter estimates for this cluster across conditions revealed a statistically significant preference for Arabic numerals, motivating future research into this area as a possible neural correlate of the VNFA. Although these coordinates have not—to our knowledge—been reported in previous explorations of the VNFA, they align closely with a cluster located at 53, -44, -12 (MNI) reported by Hannagan et al. [65]. It should be noted that areas responding preferentially to Arabic digits have also been found in the bilateral MTG, left SMG, and IFG, which further illustrates a need for examining the neural correlates of Arabic number processing. For instance, both Abboud et al. [43] as well as Amalric and Dehaene [76] demonstrated task-specific activity associated with Arabic numbers in the bilateral MTG, whereas the latter have also identified a potential neural correlate of the VNFA in the bilateral ITG. Roux et al. [77] used direct cortical electrostimulation to dissociate Arabic number reading and alphabetic script reading (both sentences and number words), finding significant interference for Arabic number reading when stimulation was applied to areas predominantly located in the left SMG, IFG, and area TE3 bordering the middle and inferior temporal gyri. The current study demonstrated activity in the left IFG, but not the left SMG and area TE3, which nevertheless should be investigated as possible sites for the VNFA. Given the condition that the right ITG only demonstrated task-based activity when subjected to a region of interest analysis, but did not serve as a region of peak-level activity in the whole-brain analyses, there is a strong case for future research to also consider the interactions of multiple distributed regions as indicative of Arabic number processing. Such a view falls well in line with the observed distribution of activity throughout the brain in the current results, and may further explain the heterogeneity of proposed locations for the VNFA when considered as a domain-specific modular system (see [42] for an overview of proposed functional regions). That is, while the response preference for Arabic numbers is observable in the currently identified candidate area of the VNFA (MNI coordinates: 42, –46, –17), this does not exclusively rule out the need for interactions with other functional areas in order to discriminate Arabic number stimuli. Yeo et al. [42] also follow this line of reasoning in arguing that the proposed VNFA in the right ITG is likely part of a larger symbolic number network, comprised additionally of the bilateral IPL, IPS, right precuneus, SPL, SFG, ACC, and IFG.

In line with our hypotheses, an atlas-based ROI analysis of the IPS (using the AIPS_1–3 probability maps in SPM Anatomy Toolbox [53]) revealed consistent activity in the right anterior area hIP1. Contrary to our predictions, only the right IPS demonstrated activity during the Arabic digit comparison task, rather than hypothesized bilateral IPS activity patterns. A recent review by Sokolowski and Ansari [78] concludes that an increasing amount of studies implicate the right IPS as the locus of magnitude processing, in line with the current results. Ansari and Dhital [79] furthermore found that left IPS activity correlated more strongly to the distance effect than numerical magnitude processing in general, which the results of this study also appear to corroborate. Moreover, Simon et al. [80] identified the left IPS as more active during calculation tasks than numerical comparison, which indicates that the right and left IPS may be functionally distinct. It should be noted that meta-analyses of brain areas subserving numerical cognition in children [81] indicate a right-hemispheric IPS dominance for numerical discrimination tasks, whereas left IPS activity is elicited by effortful, rule-based calculation (cf. [78, 80]). When considered in tandem with the distance effect, it is probable that the functional asymmetry of both intraparietal sulci exists along an *effortful–automatic* continuum of cognitive demand. This continuum ranges from the more effortful process of arithmetic to more automatized numerical discrimination skills (see Arsalidou et al. [81] for an excellent exposition of this argument), which differ in complexity depending on the relative closeness of the compared numbers. Thus, the current results may be argued to illustrate that discrimination of close numerical stimuli aligns closer with demands elicited by numerical calculation tasks (subserved primarily by the left IPS), while distant numerical stimuli depend more on automatic semantic processing accommodated by the right IPS. We suggest that the activation observed exclusively in the right IPS is indicative of a net effect, where both parametric levels of numerical discrimination engage the area as a result of conceptual task similarity, whereas the left IPS is only significantly elicited when effortful trials are observed in isolation.

The patterns of activation elicited in the amygdala were not anticipated, but may be explained as a consequence of sub-clinical mathematics anxiety (see e.g. [82]). Since the current study did not control for measures of mathematics anxiety, this interpretation necessarily remains speculative and open for future exploration. Considering, however, that arithmetic is most commonly experienced and performed with Arabic number symbols, such stimuli constitute probable catalysts of negative affective responses over less context-relevant number words and dot arrays.

### Verbal number comparison

Task-specific activation for the verbal number comparison task was contrasted with the control condition (at a threshold of *p* < .01 FWE), predominantly illustrating hypothesized patterns of activation extending through the ventral stream and left perisylvian language areas. As observed for the Arabic digit comparison task, activity was identified in area V2 of the early visual stream, further extending the discovery of visually supported nonsymbolic magnitude processing (see [30–32]) to potentially encompass symbolic numerical formats. It would be a task for future research to further address whether numerosity-sensitive neurons in area V2 respond invariably to commonly experienced representations of number.

In line with our hypotheses and the Triple Code Model [4, 47], left perisylvian language areas IFG and MTG displayed significant activation during verbal number comparison (at a threshold of *p* < .05 FWE). No activity was identified in the left STG or left SMG, as argued by Schmithorst and Douglas Brown [35] as well as Dehaene et al. [24], although task-related activation was found in the right STG. Results linking the right STG to verbally mediated numerical cognition are scarce, given the dominant view of verbal left-lateralization, although some studies have linked right STG activity to addition tasks [7]. Dehaene et al. [83] furthermore used a PET-based study to identify increased activation in the right STG when contrasting numerical comparison with multiplication. The current study identified similar patterns of activity for the Arabic and verbal comparison tasks, suggesting that this area may play an important role in format-independent symbolic number comparison. Similarly to the Arabic digit comparison task, task-specific activation was found in the right IPS (area hIP1).

The right hIP1 cluster, observed in both the Arabic and verbal number comparison tasks, demonstrated considerable overlap with the anterior (PGa) subdivision of the right angular gyrus. Being immediately bordering regions, this overlap is not surprising, and functional connectivity between these two regions and the ventrolateral prefrontal cortex (VLPFC) has been established to a significantly larger degree than for bordering IPS and AG subdivisions [84] (see also [85] for results indicating significantly stronger right-lateralized fronto-parietal connectivity in adults). A similar result can be observed in a recent study by He et al. [86], who argued that a corresponding region (albeit reported as the right temporoparietal junction) demonstrated preference to symbolic Arabic digits, as a result of engaging the bottom-up ventral attention system. This explanation aligns well with the strong functional connectivity between the right PGa/hIP1 and VLPFC, which constitute components of this network. As a brief aside, the IPS is generally regarded as part of the top-down dorsal attention system, but it is plausible that both attention systems—regardless of the specific affiliation of hIP1—operate in tandem (see e.g. [87]). Thus, the involvement of the right PGa in addition to hIP1 in symbolic number comparison tasks may be indicative of an interplay of attentional systems, both in the context of direct bottom-up perception for small symbolic quantities (as argued in [86]), as well as a goal-directed attentional process that integrates symbolic representations with the numerical magnitude code in a top-down fashion.

Beyond the exclusively right-lateralized STG activity, bilateral MTG activity was found to be elicited by the verbal number comparison task. The right MTG has previously been observed to be modulated by the numerical distance effect for Arabic numbers [88, 89], but generally elicits left-lateralized activity during verbal number processing [47]. However, it may be the case that the bilateral MTG activity found only for the verbal number comparison task is indicative of increased attentional demands to semantic information, as argued by Price et al. [90]. Although future studies should further examine these predictions, we hypothesize that the relative unconventionality of visually presented verbal number words may explain increased attentional demands associated with the interpretation of such symbolic number formats.

The observed thalamic activity associated with verbal number word comparison is implicated in Dehaene and Cohen’s [47] account of the TCM. Recent structural analyses of the fronto-parietal network of numerical cognition increasingly argue for the existence of projection fibers that link the thalamus and basal ganglia to key gray matter areas involved in numerical cognition [8]. Future research should investigate the involvement of the thalamus and general white matter substrates using suitably targeted methods, such as Diffusion Tensor Imaging.

### Nonsymbolic magnitude comparison

Task-specific activation for the nonsymbolic magnitude comparison task was contrasted with the control condition (at a threshold of *p* < .01 FWE), demonstrating a pattern of activation predominantly located within the occipital and frontal lobes. In line with previous tasks, large clusters of activity were found in visual area hOc2 (V2), as well as in area hOc3v (V3). These activity patterns correspond well to previous research by Fornaciai et al., Roggeman et al., and Cavdaroglu et al. [30–32], supporting the existence of neuronal populations sensitive to nonsymbolic representations of number in early visual areas. Given the observed activity in area hOc2 (V2) across all task–control contrasts in this study, we call for future replication attempts in order to determine the scope of visual numerosity perception.

No distinctive task-specific activation was found in the IPS (at a threshold of *p* < .05 FWE), despite the employment of an atlas-based ROI analysis for bilateral areas hIP1-3. This result not only conflicts with our hypotheses, but a majority of studies investigating the neural correlates of nonsymbolic numerical cognition (see e.g. [5, 91]). However, the involvement of early visual areas corresponds well to previously discussed research by Fornaciai et al. [30] (see also [31, 32, 92]). In this vein, Collins and colleagues [10] argue that nonsymbolic numerosity is encoded in the subcortical (prestriate) visual system, given that animals without complex cortical structures (such as homologues of the human IPS) appear able to perform numerical discrimination. This claim was evaluated experimentally, where a single-eye, monocular advantage for nonsymbolic numerical discrimination was argued to indicate the involvement of processes in visual areas preceding cortical area V1, occurring before isolated signals from each eye are combined. Despite the recent surge in studies implicating early visual areas in nonsymbolic number processing, such results are relatively uncommon. Fornaciai et al. [30] argue that this may be explained by the use of small numerosities in many previous studies, which have been shown to elicit weaker activations in number-sensitive visual areas. Notably, activity has been found to be virtually absent within the subitizing range (i.e. 1–4) [93]. These results could explain the involvement of early visual areas in the current study, given that a minimum of 13 dots were presented in each nonsymbolic trial.

It is possible that the involvement of the IPS in numerical discrimination tasks reflects associated attentional demands, rather than a semantic interpretation process of numerosity *per se*. Results corroborating such an account can be found in Cavdaroglu et al. [32], who observed activity in the occipital cortex, but no IPS activity, during passive viewing of sequentially presented nonsymbolic number arrays (see also [31]). In contrast, the active comparison of nonsymbolic number arrays and the associated response engagement resulted in predicted patterns of parietal activity, suggesting IPS activity to be indicative of response selection and comparison processes. Note, however, that the sequential stimulus presentation employed by the authors differs from the concurrent presentation format in the current study, and that several studies have reported parietal activity during passive concurrent viewing of nonsymbolic magnitude (e.g. [94, 95]). Taken together, these disparate results could be argued to suggest that concurrent observation of nonsymbolic numerical stimulus arrays may, in many cases, lead participants to compare numerical magnitude despite a lack of such task demands. Such an argument would, in our view, favor an interpretation of the IPS as chiefly associated with attentional, comparative, and response-related processes, which is plausible given that the IPS has been argued to constitute part of the dorsal attention system, and functional connectivity has specifically been established between area hIP1 (which was implicated in the symbolic tasks of the present study) and frontal ventral attentional regions [84].

The absence of IPS activity in the nonsymbolic magnitude comparison task can minimally be explained in one of two ways. A more elaborate account could suggest that symbolic numerosity discrimination, as a learned rather than phylogenetically predisposed skill, entails higher attentional demands and subsequent involvement of the IPS than nonsymbolic magnitude processing, in line with previously reported IPS involvement in processes requiring comparison, response selection, and attentional resources [32, 84]. Instead, visually mediated nonsymbolic magnitude discrimination may be possible without the involvement of the IPS. However, such an account contradicts IPS activity during passive observation of nonsymbolic magnitude arrays—critically assuming that a lack of task demands discourages participants from comparing the presented numerosities. A more parsimonious account would instead suggest that the control task, despite being presented symbolically, is more similarly coded to nonsymbolic than symbolic magnitude discrimination in the right IPS; which could explain the prevalence of left IPS activity in the parametric comparison of nonsymbolic tasks, as well as right IPS activity prevalent in symbolic trials. Lyons et al. [96] provided evidence in favor of such an explanation, arguing that the IPS processes symbolic and nonsymbolic numbers in qualitatively different fashions. In a sequentially presented stimulus-matching paradigm, participants were asked to indicate whether the second Arabic or nonsymbolic number matched the first, where a correlation in activity between similar values across both representational formats (e.g. 3 and •••) at the voxel level would indicate similar coding mechanisms in the bilateral IPS. While the IPS was found to be active for both tasks, cross-format correlations were significantly weaker than within-format correlations for both matched and differing numbers, and—contrary to within-format correlations—no significant difference was found between same and different-number correlations. Although it remains to be explored how the difference in coding of these two representational formats relates to the possible subtraction of right IPS activity in the [Nonsymbolic > Control] contrast, the upshot of this argument is the plausibility of signal subtraction for just one numerical format, as explained by the differences in coding at the voxel level for symbolic and nonsymbolic number. Nevertheless, given the unconventionality of absent IPS activity during nonsymbolic magnitude comparison in the existing literature (but see [44]), and the relative novelty of theoretical frameworks implicating visually mediated numerical perception, more research into the neural substrates of nonsymbolic magnitude processing is required.

The pattern of activity observed in the left IFG was unexpected, as this area has been argued to be part of a symbolic number processing network [42]. For instance, Roux et al. [77] found that small areas (< 1 cm^2^) within the left IFG exclusively impaired Arabic number identification and reading during electrocortical stimulation, while both number and task-dissociated words were not impacted. However, Abboud et al. [43] performed a functional connectivity analysis independently seeded from the VNFA and Visual Word Form Area (VWFA), regressing out the functional connectivity of the opposing region during analysis. A clear dissociation between VNFA activity and left IFG activity was found, indicating that left IFG activity is exclusively related to activity in the VWFA. These disparate results do not conclusively implicate the left IFG in symbolic number processing, and should be taken with caution as the current study demonstrated consistent left IFG activity across all tasks. Although future research should systematically address the role of the left IFG in numerosity processing, these results tentatively suggest that activity in this area is indicative of format-independent numerical magnitude processing, and a key component in the fronto-parietal number processing network.

### Parametric comparisons

The parametric [Close > Distant] contrasts, associated with “difficult” and “easy” trials for all conditions, revealed consistent patterns of activity in the left IPS (hIP3) and left precentral gyrus (at a threshold of *p* < .001 uncorrected). Additionally, the [Close > Distant] contrast of the nonsymbolic task resulted in activity within area PFt of the left IPL (part of the left SMG [97]). These results provide a number of outstanding questions regarding left IPS activity across task–control contrasts. For instance, it may be the case that the control task was also processed as a form of magnitude judgment. Hence, when the control condition is omitted from analysis, consistent patterns of left IPS activity are found for all tasks. These results could be explained by the existence of a spatially represented mental alphabet line, akin to the mental number line. Support for such a view can be traced to Gevers, Reynvoet, and Fias [98], who found a spatial coding effect for letters. In an experiment measuring reaction times to an order-relevant (target letter placed before or after O) and an order-irrelevant (consonant–vowel classification) letter task, Gevers et al. found that the alphabetic spatial location of a target letter influenced lateralized left and right-hand response times, where early letters of the alphabet were responded to faster with the left hand, and vice versa for later letters and the right hand. However, Zhao et al. [99] employed an ERP study to demonstrate a dissociation of spatial order and magnitude, where participants showed a reversed distance effect when learning the spatial order of meaningless symbolic stimuli rather than associating them with numerical dot arrays. These results stand in contrast to the findings of Gevers et al. [98], and it is therefore difficult to attribute the lack of left IPS activity in the current study to the control task beyond reasonable doubt. Nevertheless, magnitude processing encompasses many different properties (e.g. luminosity and physical size [100]), making it difficult to design a control condition which is both task-relevant and does not interfere with magnitude processing.

The current results strongly endorse the prediction that left IPS activity predominantly signifies a distance effect, whereas the right IPS is functionally specific to general magnitude processing [78, 79]. Similar results were obtained by Mussolin et al. [101], arguing for the progressive disengagement of left IPS and frontal regions with increasing age, whereas the right IPS was argued to support the semantic representation of numbers. Extending the argument presented by the authors, the present results imply that the left IPS may not be explicitly related to magnitude representation, but rather engaged in supportive cognitive mechanisms during numerical perception and judgment. The adult participant sample featured in this study could therefore be argued to mirror the results of Mussolin et al., where the left IPS was only recruited during putatively “difficult” numerical comparisons, whereas the right IPS was recruited during—at the very least—semantic representation of symbolic numbers.

Activity in the left precentral gyrus is common throughout the literature on numerical cognition. While it is typically attributed to task demands associated with mental arithmetic, several studies have implicated the left precentral gyrus in response selection and hand movements associated with counting [102–104]. Similarly, the bilateral supramarginal gyrus (of which area PFt is a subdivision) has regularly been implicated in numerical fact retrieval [105] and working memory processes associated with the manipulation of numerical information [106].

The behavioral results associated with task difficulty revealed differences in reaction times and accuracy for the different conditions, notably slower response times for distant (“easy”) Arabic and verbal trials. These results should be viewed with caution in light of the neurocognitive results, which fit well with previous findings showing a distance effect modulated by activity in the left IPS, where we find that close (“difficult”) trials—regardless of task—promote significant left IPS activity [79]. It should also be noted that, while a difference in reaction times for the close and distant symbolic trials does exist, the mean reaction time is largely consistent across trials whereas differences in left IPS activity are notably more striking.

### Overlapping activations across tasks

A conjunction analysis of all task–control contrasts (at a threshold of *p* < .05 FWE) revealed activation patterns resembling the recently proposed fronto-parietal network of numerical cognition [44, 50]. Activation was found to span the SPL, SFG, ACC, MCC, PCC, and the primary somatosensory cortex, bordering the previously identified key area SMA. In contrast to our predictions, no suprathreshold activity was identified in the IPS or MFG, as the former was absent from the nonsymbolic magnitude comparison task, and the latter was present only for the Arabic number comparison task. The pattern of activation found in the IFG lends credence to the tentative inclusion of this area within the fronto-parietal network model, as discussed in previous sections. Contrary to previous studies, no explicit SMA-related activity was found in this analysis. This result could be explained by recent findings indicating greater functional connectivity between the right IPS and SMA in children with mathematical disabilities [50], whereas such connectivity presently appears to be weaker in typically developed adults.

Activity in the anterior cingulate cortex (ACC) is a consistent finding throughout the literature on numerical cognition, related to magnitude processing in both format-dependent and independent contexts [44]. While the meta-analysis by Sokolowski et al. [44] did not report ACC involvement in symbolic number tasks, such patterns of activity were found for all tasks in this study. These results suggest that the ACC (as well as the middle cingulate cortex) is indeed an integral component of numerical discrimination tasks, but raise questions regarding its functional assignment. Schmithorst and Douglas Brown [35] argue that the ACC—together with prefrontal areas—is key in the selection and appropriate sequencing of numerical stimuli, extending Dehaene and Cohen’s [47] proposed direct and indirect routes of numerosity processing. This interpretation is plausible, given the observed activity in prefrontal regions SFG and IFG. The conjunction analysis additionally highlighted activity in the insula, which together with the ACC has been identified to play a significant role in goal-directed and task-specific behavior [22]. It should be noted that this interpretation is in conflict with findings by Sokolowski et al. [44] on at least two accounts, as the authors only report insula activity in nonsymbolic conditions and argue that ACC activity is representative of demand-independent magnitude processing. However, as ACC activity was persistent across all tasks, we suggest that this may indeed be indicative of task demands related to, for instance, conflict processing (e.g. in terms of spatial–numerical incongruity [107]) and attentional control processes elicited specifically by cognitive demands associated with numerical processing (see e.g. [108, 109]). Although it is plausible that the reported fronto-parietal pattern of activity represents a network of demand-independent numerical processing, future research should investigate whether this network also ought to account for components directly related to numerical and attentional task demands.

A consistent finding throughout previous analyses has been the inclusion of early visual areas. The conjunction analysis revealed activity in retinotopic areas hOc1 (V1), hOc2 (V2), and the dorsal hOc3d (V3), further corroborating the existence of a numerosity-sensitive temporo-occipital topographic map [75] and a sensitivity to format-independent numerical information in early visual areas [30].

Finally, the task–control conjunction analysis furthermore illustrated activity in the limbic system, including the hippocampus, thalamus, and basal ganglia. Recent studies have argued that the functional connectivity between structures of the limbic system and neocortical areas accurately predicts numerical learning [8], suggesting its importance in the number processing network as well as for the acquisition and deployment of number-cognitive abilities. It should be noted that Dehaene and Cohen’s [47] account of the TCM primarily implicates the left thalamus, as part of a left cortico-subcortical loop, whereas the current results highlight the consistent involvement of the right thalamus across tasks. Right thalamus activity is often associated with arithmetic-based tasks in the literature (e.g. [22]), although it is likely that the activity observed in the current study reflects the need for finger-mediated responses. Given the connection between primary somatosensory area 3b (implicated in finger stimulation; e.g. [110]) and the right-hemisphere thalamus—as part of a motor loop—through white matter fiber tracts [8], the persistent activity of these two areas across tasks could feasibly be linked to response preparation and execution. It may also be the case that this pattern of activity reflects goal-directed finger counting strategies [111], although this explanation appears unlikely as none of the tasks required explicit counting. Following this pattern of motor activity in the basal ganglia, bilateral activity was also identified in the caudate nucleus. The TCM argues for a role of the left caudate nucleus in rote arithmetic fact retrieval [22, 71] (as part of the cortico-subcortical loop employed to directly access verbal arithmetic knowledge [47]), whereas Arsalidou and Taylor [22] have argued that right caudate nucleus is deployed to sequence bottom-up, stimulus-driven numerical information and top-down, goal-directed calculation procedures in arithmetic tasks. Right caudate activity has furthermore been associated with rule-based category learning [112], the numerical distance effect during symbolic magnitude comparison tasks in children [51], and the imagining of motor actions [113]. While such results strongly suggest the involvement of the caudate nucleus in arithmetic operations (considering that arithmetic is rule-based, complex, and beneficially supported by finger counting), the prevalence of activity in this region demonstrated by the current results could rather imply the key role of a right-hemisphere motor loop during response execution. However, considering that the caudate nucleus has predominantly been observed in arithmetic-based tasks in the literature (e.g. [22, 71, 114]), the exact role of this area—and the pattern of activity described for the limbic system more generally—requires further investigation.

A conjunction analysis of the parametric contrasts (at a threshold of *p* < .05 FWE) identified the pervasive involvement of the left IPS (area hIP3), further strengthening the argument that such activity illustrates a distance effect and related, increased task demands. As previously mentioned, the spatial extent of this cluster (1 voxel) is surprising given the conservative statistical threshold applied during the analysis. It appears unlikely that such (and similar) results are purely false-positive, given the theoretical support for left hIP3 activity throughout the literature (for a subset of studies covering the IPS in such detail, see e.g. [7, 29, 42, 72, 115, 116]).

### Limitations of the study

We have identified three primary limitations of this study, which may have influenced the outcome and validity of the results. First, the design of the experimental conditions necessitated a long MRI protocol (55 minutes). This decision has the practical consequence of separating similar tasks across a relatively large stretch of time. This has been mitigated by employing a fixed task order, thereby minimizing elapsed time between similar tasks (cf. [54]), but may have been further alleviated by using a faster event-related experimental design. The use of a blocked design is, however, positive for future investigations of task-based functional connectivity (see e.g. [115]).

Second, the use of a single control task for the three numerical codes may have caused the absence of IPS activity in the nonsymbolic magnitude comparison task. Since IPS activity is common throughout the literature on nonsymbolic magnitude comparison (e.g. [24, 52]), it may be the case–as discussed earlier–that the current control task is more similarly coded to nonsymbolic magnitude discrimination in the IPS than corresponding symbolic tasks, thereby causing a subtraction of activity (cf. [96]). Future studies may benefit from investigating whether a format-matched nonsymbolic control task produces a more theoretically supported outcome.

Finally, a third potential limitation is the lack of cluster size thresholding in addition to the applied FWE correction. Although this has been discussed at length previously (see the *fMRI data analysis* section), it should be noted that we opted to err on the side of caution in order to minimize the risk for false-negative results. The attempt to investigate the neural substrates of the entire Triple Code Model should strive to provide as detailed an account as possible, in order to facilitate future exploration of relevant functional areas and networks. Therefore, we argue that it is important to account for effects which may be smaller in spatial extent, yet potentially important. The fact that a majority of these smaller clusters align well with previous literature furthermore supports this approach, and future studies as well as meta-analyses will serve to rule out potential false-positive patterns of activity (cf. [62]).

### Future lines of investigation

The results of the current study indicate a need to further investigate the bilateral activity patterns elicited for all numerical codes, in contrast to the functionally modular and lateralized original conception of the Triple Code Model [47, 71]. The complex and functionally overlapping patterns of activation associated with numerical discrimination tasks suggest the need to move towards analyses at the functional and structural network level, employing methods such as task-based and resting-state functional connectivity analyses, and the investigation of structural-anatomical brain networks using methods such as Diffusion Tensor Imaging. Moreover, these methods may provide novel insights into the cooperation between functional networks enabling numerical cognition and the emerging picture of salience, default mode, fronto-parietal, attentional, and central-executive networks (for an overview of recent advances, see [117]). It has recently been demonstrated that individuals displaying more flexible switching between the fronto-parietal and default mode networks, in both resting-state and task-based fMRI, also tend to perform with higher acuity on tasks requiring cognitive flexibility, such as the Stroop task (see e.g. [118]). The fronto-parietal network has additionally been implicated in many cognitive functions, such as action emulation, spatial attention, working memory, and decision making [119]; suggesting that this domain-general network hosts an abundance of skills needed to support number processing. It would be an interesting task for future research to investigate how these networks overlap, differ, and cooperate in order to enable numerical cognition.

## Conclusion

In this study, we have evaluated the Triple Code Model of numerical cognition [4, 47, 71] and the neural substrates subserving the the visual Arabic number form, the verbal auditory word frame, and the analog nonsymbolic magnitude representation code. Although the model—to a certain degree—correctly predicts functionally dissociated substrates associated with each code, the emerging picture of a complex and functionally overlapping fronto-parietal network of numerical cognition calls for principled revision and expansion of the TCM. In line with recent research proposing the existence of a fronto-parietal network for numerosity processing [44], a conjunction analysis across all experimental tasks illustrated potential neural substrates in the SPL, SFG, IFG, MCC, ACC, and the limbic system. These additions align closely with an updated account of the TCM, as suggested by Arsalidou and Taylor [22].

Although we were not able to localize the Visual Number Form Area in line with previously reported cytoarchitectonic coordinates, we did identify a cluster which exhibited preferential responses to Arabic numbers within the right ITG (although its peak was located in a larger cluster spanning the right MTG), as demonstrated by previous research [42]. We suggest that future research investigates this specific cluster, as well as the roles of the bilateral middle temporal and left inferior frontal gyri in Arabic number processing (see [76, 77]). However, future studies should also consider the possibility that Arabic number processing is not subserved by single functional areas, but rather that the identified VNFA candidate area may be part of a larger network of regions which express selectivity for such stimuli. Following previous research, we identified neural substrates associated with verbal number word processing in the left perisylvian language areas [35], with the notable exception of the left STG and SMG. The finding of right STG activity associated with the verbal number discrimination task suggests the importance of this region for format-independent symbolic number comparison [83], as evidenced by its prevalence for both Arabic and verbal stimuli. Together with right STG activity, we observed activity in areas typically associated with calculation tasks, such as the left caudate nucleus and precentral gyrus [22, 71, 102–104]. These results suggest the importance of further evaluating the roles of functional substrates and their purported specificity for numerical discrimination and calculation tasks. The exclusive presence of right IPS activity during symbolic numerical tasks poses an interesting challenge to previous research (see e.g. [22]), and could imply the existence of visual nonsymbolic magnitude processing in adult populations [30–32]. Neural correlates of nonsymbolic magnitude discrimination were observed in visual areas V2 and V3, while only left IPS activity was found when contrasting putatively difficult and easy trials, as explained by the distance effect.

We argue that the large participant sample featured in this study, as well as the use of parametric contrasts to explicitly specify distinct patterns of activity, provides a strong foundation for further structural and functional analyses of neural substrates associated with the Triple Code Model. We suggest that future research attempts to replicate these results and details the functional characteristics of components associated with the Triple Code Model, which may provide insight into neurocognitive disabilities such as Developmental Dyscalculia.

## Supporting information

S1 Fig Response time and accuracy distribution. Significant differences in reaction times and response accuracy are illustrated across tasks. Close and distant numerical trials are indicated by [C] and [D] respectively. (A) Parametric contrasts of response time (left) and accuracy (right). (B) Cross-condition contrasts of response time (left) and accuracy (right).
(TIF)

## References

[1] Parsons S, Bynner J. Does numeracy matter more?; 2005. Available from: http://eprints.ioe.ac.uk/4758/1/parsons2006does.pdf [cited 2017-10-19].

[2] Gross J, Hudson C, Price D. The long term costs of numeracy difficulties; 2009. Available from: http://www.northumberland.gov.uk/WAMDocuments/C9AEE344-23BE-4450-B9AA-41AAA891563A_1_0.pdf?nccredirect=1 [cited 2017-10-10].

[3] Kaufmann L, Wood G, Rubinsten O, Avishai H. Meta-Analyses of Developmental fMRI Studies Investigating Typical and Atypical Trajectories of Number Processing and Calculation. Developmental Neuropsychology. 2011;36(6):736–787. doi: 10.1080/87565641.2010.549884 10.1080/87565641.2010.549884 21761997

[4] Dehaene S. Varieties of numerical abilities. Cognition. 1992;44(1–2):1–42. doi: 10.1016/0010-0277(92)90049-N 1511583 10.1016/0010-0277(92)90049-n

[5] Nieder A. The neuronal code for number. Nature Reviews Neuroscience. 2016;17(6):366–382. doi: 10.1038/nrn.2016.40 27150407 10.1038/nrn.2016.40

[6] Peters L, Polspoel B, Op de Beeck H, De Smedt B. Brain activity during arithmetic in symbolic and non-symbolic formats in 9–12 year old children. Neuropsychologia. 2016;86:19–28. doi: 10.1016/j.neuropsychologia.2016.04.001 27044845 10.1016/j.neuropsychologia.2016.04.001

[7] Klein E, Suchan J, Moeller K, Karnath HO, Knops A, Wood G, et al Considering structural connectivity in the triple code model of numerical cognition: differential connectivity for magnitude processing and arithmetic facts. Brain Structure and Function. 2016;221(2):979–995. doi: 10.1007/s00429-014-0951-1 25432772 10.1007/s00429-014-0951-1

[8] Moeller K, Willmes K, Klein E. A review on functional and structural brain connectivity in numerical cognition. Frontiers in Human Neuroscience. 2015;9:227 doi: 10.3389/fnhum.2015.00227 26029075 10.3389/fnhum.2015.00227 PMC4429582

[9] Brannon EM, Jordan KE, Jones SM. Behavioral Signatures of Numerical Discrimination In: Platt ML, Ghazanfar AA, editors. Primate Neuroethology. Oxford: Oxford University Press; 2010 p. 144–159.

[10] Collins E, Park J, Behrmann M. Numerosity representation is encoded in human subcortex. PNAS. 2017;114(14):E2806–E2815. doi: 10.1073/pnas.1613982114 28320968 10.1073/pnas.1613982114 PMC5389276

[11] Starkey P, Cooper R. Perception of Numbers by Human Infants. Science. 1980;210(4473):1033–1035. doi: 10.1126/science.7434014 7434014 10.1126/science.7434014

[12] Gallistel CR, Gelman R. Non-verbal numerical cognition: From reals to integers. Trends in Cognitive Sciences. 2000;4(2):59–65. doi: 10.1016/S1364-6613(99)01424-2 10652523 10.1016/s1364-6613(99)01424-2

[13] Halberda J, Mazzocco MMM, Feigenson L. Individual differences in non-verbal number acuity correlate with maths achievement. Nature. 2008;455(7213):665 doi: 10.1038/nature07246 18776888 10.1038/nature07246

[14] Piazza M. Neurocognitive start-up tools for symbolic number representations. Trends in Cognitive Sciences. 2010;14(12):542–551. doi: 10.1016/j.tics.2010.09.008 21055996 10.1016/j.tics.2010.09.008

[15] Dehaene S. The number sense: how the mind creates mathematics. New York: Oxford University Press; 2011.

[16] Hyde DC. Two systems of non-symbolic numerical cognition. Frontiers in Human Neuroscience. 2011;5:150 doi: 10.3389/fnhum.2011.00150 22144955 10.3389/fnhum.2011.00150 PMC3228256

[17] Libertus ME, Feigenson L, Halberda J. Preschool acuity of the approximate number system correlates with school math ability. Developmental Science. 2011;14(6):1292–300. doi: 10.1111/j.1467-7687.2011.01080.x 22010889 10.1111/j.1467-7687.2011.01080.x PMC3338171

[18] Xu F. Numerosity discrimination in infants: Evidence for two systems of representations. Cognition. 2003;89:B15–B25. doi: 10.1016/S0010-0277(03)00050-7 12893126 10.1016/s0010-0277(03)00050-7

[19] Rouder J, Geary D. Children’s Cognitive Representation of the Mathematical Number Line. Developmental Science. 2014;17(4):525–536. doi: 10.1111/desc.12166 24796511 10.1111/desc.12166 PMC4439402

[20] Kaufmann L, Vogel SE, Wood G, Kremser C, Schocke M, Zimmerhackl LB, et al A developmental fMRI study of nonsymbolic numerical and spatial processing. Cortex; a Journal Devoted to the Study of the Nervous System and Behavior. 2008;44(4):376–85. doi: 10.1016/j.cortex.2007.08.003 18387568 10.1016/j.cortex.2007.08.003

[21] Ansari D. Effects of development and enculturation on number representation in the brain. Nature Reviews Neuroscience. 2008;9(4):278–91. doi: 10.1038/nrn2334 18334999 10.1038/nrn2334

[22] Arsalidou M, Taylor MJ. Is 2+2 = 4? Meta-analyses of brain areas needed for numbers and calculations. NeuroImage. 2011;54(3):2382–2393. doi: 10.1016/j.neuroimage.2010.10.009 20946958 10.1016/j.neuroimage.2010.10.009

[23] Butterworth B, Varma S, Laurillard D. Dyscalculia: From brain to education. Science. 2011;332(6033):1049–1053. doi: 10.1126/science.1201536 21617068 10.1126/science.1201536

[24] Dehaene S, Piazza M, Pinel P, Cohen L. Three Parietal Circuits for Number Processing. Cognitive Neuropsychology. 2003;20(3–6):487–506. doi: 10.1080/02643290244000239 20957581 10.1080/02643290244000239

[25] Moyer RS, Landauer TK. Time required for Judgements of Numerical Inequality. Nature. 1967;215(5109):1519–20. doi: 10.1038/2151519a0 6052760 10.1038/2151519a0

[26] Dehaene S. The neural basis of the Weber-Fechner law: a logarithmic mental number line. Trends in Cognitive Sciences. 2003;7(4):145–147. doi: 10.1016/S1364-6613(03)00055-X 12691758 10.1016/s1364-6613(03)00055-x

[27] Van Opstal F, Gevers W, De Moor W, Verguts T. Dissecting the symbolic distance effect: Comparison and priming effects in numerical and nonnumerical orders. Psychonomic Bulletin & Review. 2008;15(2):419–425. doi: 10.3758/PBR.15.2.419 18488662 10.3758/pbr.15.2.419

[28] Bulthé J, De Smedt B, Op de Beeck HP. Visual Number Beats Abstract Numerical Magnitude: Format-dependent Representation of Arabic Digits and Dot Patterns in Human Parietal Cortex. Journal of Cognitive Neuroscience. 2015;27(7):1376–1387. doi: 10.1162/jocn_a_00787 25633646 10.1162/jocn_a_00787

[29] Bulthé J, De Smedt B, Op de Beeck HP. Format-dependent representations of symbolic and non-symbolic numbers in the human cortex as revealed by multi-voxel pattern analyses. NeuroImage. 2014;87:311–322. doi: 10.1016/j.neuroimage.2013.10.049 24201011 10.1016/j.neuroimage.2013.10.049

[30] Fornaciai M, Brannon EM, Woldorff MG, Park J. Numerosity processing in early visual cortex. NeuroImage. 2017;157:429–438. doi: 10.1016/j.neuroimage.2017.05.069 28583882 10.1016/j.neuroimage.2017.05.069 PMC6697050

[31] Roggeman C, Santens S, Fias W, Verguts T. Stages of Nonsymbolic Number Processing in Occipitoparietal Cortex Disentangled by fMRI Adaptation. Journal of Neuroscience. 2011;31(19):7168–7173. doi: 10.1523/JNEUROSCI.4503-10.2011 21562280 10.1523/JNEUROSCI.4503-10.2011 PMC6703200

[32] Cavdaroglu S, Katz C, Knops A. Dissociating estimation from comparison and response eliminates parietal involvement in sequential numerosity perception. NeuroImage. 2015;116:135–148. doi: 10.1016/j.neuroimage.2015.04.019 25887262 10.1016/j.neuroimage.2015.04.019

[33] Park J, Dewind NK, Woldorff MG, Brannon EM. Rapid and Direct Encoding of Numerosity in the Visual Stream. Cerebral Cortex. 2016;26(2):748–763. doi: 10.1093/cercor/bhv017 25715283 10.1093/cercor/bhv017 PMC4712802

[34] Dewind NK, Adams GK, Platt ML, Brannon EM. Modeling the approximate number system to quantify the contribution of visual stimulus features. Cognition. 2015;142:247–265. doi: 10.1016/j.cognition.2015.05.016 26056747 10.1016/j.cognition.2015.05.016 PMC4831213

[35] Schmithorst VJ, Douglas Brown R. Empirical validation of the triple-code model of numerical processing for complex math operations using functional MRI and group Independent Component Analysis of the mental addition and subtraction of fractions. NeuroImage. 2004;22(3):1414–20. doi: 10.1016/j.neuroimage.2004.03.021 15219612 10.1016/j.neuroimage.2004.03.021

[36] Price GR, Ansari D. Symbol processing in the left angular gyrus: Evidence from passive perception of digits. NeuroImage. 2011;57(3):1205–1211. doi: 10.1016/j.neuroimage.2011.05.035 21620978 10.1016/j.neuroimage.2011.05.035

[37] Jefferies E, Bateman D, Lambon Ralph MA. The role of the temporal lobe semantic system in number knowledge: evidence from late-stage semantic dementia. Neuropsychologia. 2005;43(6):887–905. doi: 10.1016/j.neuropsychologia.2004.09.009 15716160 10.1016/j.neuropsychologia.2004.09.009

[38] Cipolotti L, Warrington EK, Butterworth B. Selective impairment in manipulating Arabic numerals. Cortex; a Journal Devoted to the Study of the Nervous System and Behavior. 1995;31(1):73–86. doi: 10.1016/S0010-9452(13)80106-2 7540123 10.1016/s0010-9452(13)80106-2

[39] Fayol M, Seron X. In: Campbell DJI, editor. About numerical representations: insights from neuropsychological, experimental and developmental studies,. New York: Psychology Press; 2005 p. 3–22.

[40] Shum J, Hermes D, Foster BL, Dastjerdi M, Rangarajan V, Winawer J, et al A Brain Area for Visual Numerals. The Journal of Neuroscience. 2013;33(16):6709–6715. doi: 10.1523/JNEUROSCI.4558-12.2013 23595729 10.1523/JNEUROSCI.4558-12.2013 PMC3970733

[41] Grotheer M, Ambrus GG, Kovács G. Causal evidence of the involvement of the number form area in the visual detection of numbers and letters. NeuroImage. 2016;132:314–319. doi: 10.1016/j.neuroimage.2016.02.069 26940623 10.1016/j.neuroimage.2016.02.069

[42] Yeo DJ, Wilkey ED, Price GR. The search for the number form area: A functional neuroimaging meta-analysis. Neuroscience and Biobehavioral Reviews. 2017;78:145–160. doi: 10.1016/j.neubiorev.2017.04.027 28467892 10.1016/j.neubiorev.2017.04.027

[43] Abboud S, Maidenbaum S, Dehaene S, Amedi A. A number-form area in the blind. Nature Communications. 2015;6(6026):1–9. 10.1038/ncomms7026 PMC4338545 25613599

[44] Sokolowski HM, Fias W, Mousa A, Ansari D. Common and distinct brain regions in both parietal and frontal cortex support symbolic and nonsymbolic number processing in humans: A functional neuroimaging meta-analysis. NeuroImage. 2016;146(2016):376–394. doi: 10.1016/j.neuroimage.2016.10.028 27769786 10.1016/j.neuroimage.2016.10.028

[45] Polk TA, Stallcup M, Aguirre GK, Alsop DC, D’Esposito M, Detre JA, et al Neural specialization for letter recognition. Journal of Cognitive Neuroscience. 2002;14(2):145–159. doi: 10.1162/089892902317236803 11970782 10.1162/089892902317236803

[46] Cohen L, Dehaene S. Specialization within the ventral stream: the case for the visual word form area. NeuroImage. 2004;22(1):466–476. doi: 10.1016/j.neuroimage.2003.12.049 15110040 10.1016/j.neuroimage.2003.12.049

[47] Dehaene S, Cohen L. Cerebral Pathways for Calculation: Double Dissociation between Rote Verbal and Quantitative Knowledge of Arithmetic. Cortex. 1997;33(2):219–250. doi: 10.1016/S0010-9452(08)70002-9 9220256 10.1016/s0010-9452(08)70002-9

[48] Skagerlund K, Karlsson T, Träff U. Magnitude processing in the brain: An fMRI study of time, space, and numerosity as a shared cortical system. Frontiers in Human Neuroscience. 2016;10(500):1–12. 27761110 10.3389/fnhum.2016.00500 PMC5050204

[49] Walsh V. A theory of magnitude: common cortical metrics of time, space and quantity. Trends in Cognitive Sciences. 2003;7(11):483–488. doi: 10.1016/j.tics.2003.09.002 14585444 10.1016/j.tics.2003.09.002

[50] Jolles D, Ashkenazi S, Kochalka J, Evans T, Richardson J, Rosenberg-Lee M, et al Parietal hyper-connectivity, aberrant brain organization, and circuit-based biomarkers in children with mathematical disabilities. Developmental Science. 2016;19(4):613–631. doi: 10.1111/desc.12399 26874919 10.1111/desc.12399 PMC4945407

[51] Ansari D, Garcia N, Lucas E, Hamon K, Dhital B. Neural correlates of symbolic number processing in children and adults. NeuroReport. 2005;16(16):1769–1773. doi: 10.1097/01.wnr.0000183905.23396.f1 16237324 10.1097/01.wnr.0000183905.23396.f1

[52] Nieder A, Dehaene S. Representation of number in the brain. Annual Review of Neuroscience. 2009;32:185–208. doi: 10.1146/annurev.neuro.051508.135550 19400715 10.1146/annurev.neuro.051508.135550

[53] Eickhoff S, Stephan KE, Mohlberg H, Grefkes C, Fink GR, Amunts K, et al A new SPM toolbox for combining probabilistic cytoarchitectonic maps and functional imaging data. NeuroImage. 2005;25(4):1325–1335. doi: 10.1016/j.neuroimage.2004.12.034 15850749 10.1016/j.neuroimage.2004.12.034

[54] Henson R. Efficient Experimental Design for fMRI In: Friston K, editor. Statistical Parametric Mapping: The Analysis of Functional Brain Images. Oxford: Academic Press; 2007 p. 193–210.

[55] Leibovich T, Katzin N, Harel M, Henik A. From ‘sense of number’ to ‘sense of magnitude’—The role of continuous magnitudes in numerical cognition. Behavioral and Brain Sciences. 2016; p. 1–62. 27530053 10.1017/S0140525X16000960

[56] Mix KS, Huttenlocher J, Levine SC. Multiple Cues for Quantification in Infancy: Is Number One of Them? Psychological Bulletin. 2002;128(2):278–294. 11931520 10.1037/0033-2909.128.2.278

[57] Libertus ME, Starr A, Brannon EM. Number Trumps Area for 7-Month-Old Infants. Developmental Psychology. 2014;50(1):108–112. doi: 10.1037/a0032986 23647413 10.1037/a0032986 PMC3796133

[58] Cohen L, Martinaud O, Lemer C, Lehéricy S, Samson Y, Obadia M, et al Visual Word Recognition in the Left and Right Hemispheres: Anatomical and Functional Correlates of Peripheral Alexias. Cerebral Cortex. 2003;13(12):1313–1333. doi: 10.1093/cercor/bhg079 14615297 10.1093/cercor/bhg079

[59] James KH, James TW, Jobard G, Wong ACN, Gauthier I. Letter processing in the visual system: different activation patterns for single letters and strings. Cognitive, Affective & Behavioral Neuroscience. 2005;5(4):452–466. doi: 10.3758/CABN.5.4.452 10.3758/cabn.5.4.452 16541814

[60] Dufor O, Rapp B. Letter representations in writing: an fMRI adaptation approach. Frontiers in Psychology. 2013;4:781 doi: 10.3389/fpsyg.2013.00781 24194724 10.3389/fpsyg.2013.00781 PMC3809555

[61] Wimmer H, Ludersdorfer P, Richlan F, Kronbichler M. Visual Experience Shapes Orthographic Representations in the Visual Word Form Area. Psychological Science. 2016;27(9):1240–1248. doi: 10.1177/0956797616657319 27435995 10.1177/0956797616657319 PMC5017316

[62] Liebermann MD, Cunningham WA. Type I and Type II error concerns in fMRI research: re-balancing the scale. Social Cognitive and Affective Neuroscience. 2009;4(1):423–428. doi: 10.1093/scan/nsp052 20035017 10.1093/scan/nsp052 PMC2799956

[63] Scheperjans F, Eickhoff SB, Hömke L, Mohlberg H, Harmann K, Amunts K, et al Probabilistic maps, morphometry, and variability of cytoarchitectonic areas in the human superior parietal cortex. Cerebral Cortex. 2008;18(9):2141–2157. doi: 10.1093/cercor/bhm241 18245042 10.1093/cercor/bhm241 PMC3140197

[64] Hermes D, Rangarajan V, Foster BL, King JR, Kasikci I, Miller KJ, et al Electrophysiological Responses in the Ventral Temporal Cortex During Reading of Numerals and Calculation. Cerebral Cortex. 2015;27(1):567–575. 10.1093/cercor/bhv250 PMC5939218 26503267

[65] Hannagan T, Amedi A, Cohen L, Dehaene-Lambertz G, Dehaene S. Origins of the specialization for letters and numbers in ventral occipitotemporal cortex. Trends in Cognitive Sciences. 2015;19(7):374–382. doi: 10.1016/j.tics.2015.05.006 26072689 10.1016/j.tics.2015.05.006

[66] Park J, Hebrank A, Polk TA, Park C. Neural Dissociation of Number from Letter Recognition and Its Relationship to Parietal Numerical Processing. Journal of Cognitive Neuroscience. 2012;24(1):39–50. doi: 10.1162/jocn_a_00085 21736455 10.1162/jocn_a_00085 PMC3357212

[67] Morosan P, Schleicher A, Amunts K, Zilles K. Multimodal architectonic mapping of human superior temporal gyrus. Anatomy and Embryology. 2005;210(5–6):401–406. doi: 10.1007/s00429-005-0029-1 16170539 10.1007/s00429-005-0029-1

[68] Caspers S, Geyer S, Schleicher A, Mohlberg H, Amunts K, Zilles K. The human inferior parietal cortex: cytoarchitectonic parcellation and interindividual variability. NeuroImage. 2006;33(2):430–448. doi: 10.1016/j.neuroimage.2006.06.054 16949304 10.1016/j.neuroimage.2006.06.054

[69] Cabeza R, Nyberg L. Imaging cognition II: An empirical review of 275 PET and fMRI studies. Journal of Cognitive Neuroscience. 2000;12(1):1–47. doi: 10.1162/08989290051137585 10769304 10.1162/08989290051137585

[70] Piazza M, Izard V, Pinel P, Le Bihan D, Dehaene S. Tuning curves for approximate numerosity in the human intraparietal sulcus. Neuron. 2004;44(3):547–555. doi: 10.1016/j.neuron.2004.10.014 15504333 10.1016/j.neuron.2004.10.014

[71] Dehaene S, Cohen L. Towards an anatomical and functional model of number processing. Mathematical Cognition. 1995;1:83–120.

[72] Price GR, Yeo DJ, Wilkey ED, Cutting LE. Prospective relations between resting-state connectivity of parietal subdivisions and arithmetic competence. Developmental Cognitive Neuroscience. In press 2017;. doi: 10.1016/j.dcn.2017.02.006 28268177 10.1016/j.dcn.2017.02.006 PMC5568461

[73] Menon V, Uddin LQ. Saliency, switching, attention and control: a network model of insula function. Brain Structure and Function. 2010;214(5–6):655–667. doi: 10.1007/s00429-010-0262-0 20512370 10.1007/s00429-010-0262-0 PMC2899886

[74] Cloutman LL. Interaction between dorsal and ventral processing streams: Where, when and how? Brain and Language. 2013;127(2):251–263. doi: 10.1016/j.bandl.2012.08.003 22968092 10.1016/j.bandl.2012.08.003

[75] Harvey BM, Dumoulin SO. A network of topographic numerosity maps in human association cortex. Nature Human Behaviour. 2017;4:1–9.

[76] Amalric M, Dehaene S. Origins of the brain networks for advanced mathematics in expert mathematicians. Proceedings of the National Academy of Sciences. 2016;113(18):4909 doi: 10.1073/pnas.1603205113 10.1073/pnas.1603205113 PMC4983814 27071124

[77] Roux FE, Lubrano V, Lauwers-Cances V, Giussani C, Démonet JF. Cortical areas involved in Arabic number reading. Neurology. 2008;70(3):210–217. doi: 10.1212/01.wnl.0000297194.14452.a0 18195265 10.1212/01.wnl.0000297194.14452.a0

[78] Sokolowski HM, Ansari D. In: Henik A, editor. Symbolic and Nonsymbolic Representations of Number in the Human Parietal Cortex: A Review of the State-of-the-Art, Outstanding Questions and Future Directions. Boston, MA: Elsevier; 2016 p. 327–350.

[79] Ansari D, Dhital B. Age-related Changes in the Activation of the Intraparietal Sulcus during Nonsymbolic Magnitude Processing: An Event-related Functional Magnetic Resonance Imaging Study. Journal of Cognitive Neuroscience. 2006;18(11):1820–1828. doi: 10.1162/jocn.2006.18.11.1820 17069473 10.1162/jocn.2006.18.11.1820

[80] Simon O, Mangin JF, Cohen L, Le Bihan D, Dehaene S. Topographical layout of hand, eye, calculation and language-related areas in the human parietal lobe. Neuron. 2002;33:475–487. doi: 10.1016/S0896-6273(02)00575-5 11832233 10.1016/s0896-6273(02)00575-5

[81] Arsalidou M, Pawliw-Levac M, Sadeghi M, Pascual-Leone J. Brain areas associated with numbers and calculations in children: Meta-analyses of fMRI studies. Developmental Cognitive Neuroscience. In press 2017;. doi: 10.1016/j.dcn.2017.08.002 28844728 10.1016/j.dcn.2017.08.002 PMC6969084

[82] Dowker A, Sarkar A, Looi CY. Mathematics Anxiety: What Have We Learned in 60 Years? Frontiers in Psychology. 2016;7:508 doi: 10.3389/fpsyg.2016.00508 27199789 10.3389/fpsyg.2016.00508 PMC4842756

[83] Dehaene S, Tzourio N, Frak V, Raynaud L, Cohen L, Mehler J, et al Cerebral activations during number multiplication and comparison: A PET study. Neuropsychologia. 1996;34:1097–1106. doi: 10.1016/0028-3932(96)00027-9 8904747 10.1016/0028-3932(96)00027-9

[84] Uddin LQ, Supekar K, Amin H, Rykhlevskaia E, Nguyen DA, Greicius MD, et al Dissociable connectivity within human angular gyrus and intraparietal sulcus: Evidence from functional and structural connectivity. Cerebral Cortex. 2010;20(11):2636–2646. doi: 10.1093/cercor/bhq011 20154013 10.1093/cercor/bhq011 PMC2951845

[85] Emerson RW, Cantlon JF. Early math achievement and functional connectivity in the fronto-parietal network. Developmental Cognitive Neuroscience. 2012;2(S1):S139–S151. doi: 10.1016/j.dcn.2011.11.003 22682903 10.1016/j.dcn.2011.11.003 PMC3375498

[86] He L, Zuo Z, Chen L, Humphreys G. Effects of Number Magnitude and Notation at 7T: Separating the Neural Response to Small and Large, Symbolic and Nonsymbolic Number. Cerebral Cortex. 2014;24(8):2199–2209. doi: 10.1093/cercor/bht074 23535179 10.1093/cercor/bht074

[87] Corbetta M, Shulman GL. Control of goal-directed and stimulus-driven attention in the brain. Nature Reviews Neuroscience. 2002;3:201–215. doi: 10.1038/nrn755 11994752 10.1038/nrn755

[88] Pinel P, Dehaene S, Rivière D, LeBihan D. Modulation of Parietal Activation by Semantic Distance in a Number Comparison Task. NeuroImage. 2001;14(5):1013–1026. doi: 10.1006/nimg.2001.0913 11697933 10.1006/nimg.2001.0913

[89] Cohen Kadosh R, Henik A, Rubinsten O, Mohr H, Dori H, van de Ven V, et al Are numbers special? The comparison systems of the human brain investigated by fMRI. Neuropsychologia. 2005;43(9):1238–1248. doi: 10.1016/j.neuropsychologia.2004.12.017 15949508 10.1016/j.neuropsychologia.2004.12.017

[90] Price CJ, Wise RJS, Watson JDG, Patterson K, Howard D, Frackowiak RSJ. Brain activity during reading: The effects of exposure duration and task. Brain. 1994;117:1255–1269. doi: 10.1093/brain/117.6.1255 7820564 10.1093/brain/117.6.1255

[91] Leibovich T, Ansari D. The symbol-grounding problem in numerical cognition: A review of theory, evidence, and outstanding questions. Canadian Journal of Experimental Psychology. 2016;70(1):12–23. doi: 10.1037/cep0000070 26913782 10.1037/cep0000070

[92] McCaskey U, von Aster M, O’Gorman Tuura R, Kucian K. Adolescents with Developmental Dyscalculia Do Not Have a Generalized Magnitude Deficit—Processing of Discrete and Continuous Magnitudes. Frontiers in Human Neuroscience. 2017;11(102):1–19. 28373834 10.3389/fnhum.2017.00102 PMC5357648

[93] Fornaciai M, Park J. Distinct Neural Signatures for Very Small and Very Large Numerosities. Frontiers in Human Neuroscience. 2017;11(January):1–14. 28197086 10.3389/fnhum.2017.00021 PMC5282473

[94] Ansari D, Dhital B, Siong SC. Parametric effects of numerical distance on the intraparietal sulcus during passive viewing of rapid numerosity changes. Brain Research. 2006;1067(1):181–188. doi: 10.1016/j.brainres.2005.10.083 16359648 10.1016/j.brainres.2005.10.083

[95] Piazza M, Pinel P, Le Bihan D, Dehaene S. A Magnitude Code Common to Numerosities and Number Symbols in Human Intraparietal Cortex. Neuron. 2007;53(2):293–305. doi: 10.1016/j.neuron.2006.11.022 17224409 10.1016/j.neuron.2006.11.022

[96] Lyons IM, Ansari D, Beilock SL. Qualitatively Different Coding of Symbolic and Nonsymbolic Numbers in the Human Brain. Human Brain Mapping. 2015;36:475–488. doi: 10.1002/hbm.22641 25238646 10.1002/hbm.22641 PMC6869776

[97] Grabner RH, Reishofer G, Koschutnig K, Ebner F. Brain Correlates of Mathematical Competence in Processing Mathematical Representations. Frontiers in Human Neuroscience. 2011;5:1–11. 22069387 10.3389/fnhum.2011.00130 PMC3208209

[98] Gevers W, Reynvoet B, Fias W. The mental representation of ordinal sequences is spatially organized. Cognition. 2003;87(3):87–95. doi: 10.1016/S0010-0277(02)00234-2 10.1016/s0010-0277(02)00234-2 12684205

[99] Zhao H, Chen C, Zhang H, Zhou X, Mei L, Chen C, et al Is Order the Defining Feature of Magnitude Representation? An ERP Study on Learning Numerical Magnitude and Spatial Order of Artificial Symbols. PLoS One. 2012;7(11):49565 doi: 10.1371/journal.pone.0049565 10.1371/journal.pone.0049565 PMC3501518 23185363

[100] Mussolin C, Martin R, Schiltz C. Relationships between number and space processing in adults with and without dyscalculia. Acta Psychologica. 2011;138(1):193–203. doi: 10.1016/j.actpsy.2011.06.004 21802651 10.1016/j.actpsy.2011.06.004

[101] Mussolin C, Noël MP, Pesenti M, Grandin C, De Volder AG. Neural correlates of the numerical distance effect in children. Frontiers in Psychology. 2013;4(OCT):1–9. 24151473 10.3389/fpsyg.2013.00663 PMC3798761

[102] Cohen Kadosh R, Walsh V. Numerical representation in the parietal lobes: abstract or not abstract? Behavioral and Brain Sciences. 2009;32(3–4):313–373. doi: 10.1017/S0140525X09990938 19712504 10.1017/S0140525X09990938

[103] Venkatraman V, Ansari D, Chee MWL. Neural correlates of symbolic and non-symbolic arithmetic. Neuropsychologia. 2005;43(5):744–753. doi: 10.1016/j.neuropsychologia.2004.08.005 15721187 10.1016/j.neuropsychologia.2004.08.005

[104] Sato M, Cattaneo L, Rizzolatti G, Gallese V. Numbers within Our Hands: Modulation of Corticospinal Excitability of Hand Muscles during Numerical Judgment. Journal of Cognitive Neuroscience. 2007;19(4):684–693. doi: 10.1162/jocn.2007.19.4.684 17381258 10.1162/jocn.2007.19.4.684

[105] Cabeza R, Ciaramelli E, Moscovitch M. Cognitive Contributions of the Ventral Parietal Cortex: An Integrative Theoretical Account. Trends in Cognitive Sciences. 2012;16(6):338–352. doi: 10.1016/j.tics.2012.04.008 22609315 10.1016/j.tics.2012.04.008 PMC3367024

[106] Chang TT, Metcalfe AWS, Padmanabhan A, Chen T, Menon V. Heterogeneous and nonlinear development of human posterior parietal cortex function. NeuroImage. 2016;126(2016):184–195. doi: 10.1016/j.neuroimage.2015.11.053 26655682 10.1016/j.neuroimage.2015.11.053

[107] Gevers W, Lammertyn J, Notebaert W, Verguts T, Fias W. Automatic response activation of implicit spatial information: Evidence from the SNARC effect. Acta Psychologica. 2006;122(3):221–233. doi: 10.1016/j.actpsy.2005.11.004 16423320 10.1016/j.actpsy.2005.11.004

[108] Szűcs D, Soltész F. Functional definition of the N450 event-related brain potential marker of conflict processing: a numerical Stroop study. BMC Neuroscience. 2012;13(35):1–14. 22452924 10.1186/1471-2202-13-35 PMC3383462

[109] Kaufmann L, Koppelstaetter F, Delazer M, Siedentopf C, Rhomberg P, Golaszewski S, et al Neural correlates of distance and congruity effects in a numerical Stroop task: an event-related fMRI study. NeuroImage. 2005;25(3):888–898. doi: 10.1016/j.neuroimage.2004.12.041 15808989 10.1016/j.neuroimage.2004.12.041

[110] Schweizer R, Voit D, Frahm J. Finger representations in human primary somatosensory cortex as revealed by high-resolution functional MRI of tactile stimulation. NeuroImage. 2008;42(1):28–35. doi: 10.1016/j.neuroimage.2008.04.184 18550386 10.1016/j.neuroimage.2008.04.184

[111] Krinzinger H, Koten JW, Horoufchin H, Kohn N, Arndt D, Sahr K, et al The role of finger representations and saccades for number processing: an fMRI study in children. Frontiers in Psychology. 2011;2:373 doi: 10.3389/fpsyg.2011.00373 22203810 10.3389/fpsyg.2011.00373 PMC3244143

[112] Ashby FG, Spiering BJ. The Neurobiology of Category Learning. Behavioral and Cognitive Neuroscience Reviews. 2004;3(2):204–210. doi: 10.1177/1534582304270782 10.1177/1534582304270782 15537987

[113] Gerardin E, Sirigu A, Lehéricy S, Poline JB, Gaymard B, Marsault C, et al Partially overlapping neural networks for real and imagined hand movements. Cerebral Cortex. 2000;10(11):1093–1104. doi: 10.1093/cercor/10.11.1093 11053230 10.1093/cercor/10.11.1093

[114] Bloechle J, Huber S, Bahnmueller J, Rennig J, Willmes K, Cavdaroglu S, et al Fact Learning in Complex Arithmetic—-The Role of the Angular Gyrus Revisited. Human Brain Mapping. 2016;37(9):3061–3079. doi: 10.1002/hbm.23226 27130734 10.1002/hbm.23226 PMC6867278

[115] Park J, Park DC, Polk TA. Parietal Functional Connectivity in Numerical Cognition. Cerebral Cortex. 2013;23(9):2127–2135. doi: 10.1093/cercor/bhs193 22784605 10.1093/cercor/bhs193 PMC3729197

[116] Rosenberg-Lee M, Ashkenazi CT S, Young CB, Geary DC, Menon V. Brain hyper-connectivity and operation-specific deficits during arithmetic problem solving in children with developmental dyscalculia. Developmental Science. 2015;18(3):351–372. doi: 10.1111/desc.12216 25098903 10.1111/desc.12216 PMC4320038

[117] Park HJ, Friston K. Structural and Functional Brain Networks: From Connections to Cognition. Science. 2013;342(6158):1238411 doi: 10.1126/science.1238411 24179229 10.1126/science.1238411

[118] Douw L, Wakeman DG, Tanaka N, Liu H, Stufflebeam SM. State-dependent variability of dynamic functional connectivity between frontoparietal and default networks relates to cognitive flexibility. Neuroscience. 2016;339:12–21. doi: 10.1016/j.neuroscience.2016.09.034 27687802 10.1016/j.neuroscience.2016.09.034 PMC5635855

[119] Ptak R, Schnider A, Fellrath J. The Dorsal Frontoparietal Network: A Core System for Emulated Action. Trends in Cognitive Sciences. 2017;21(8):589–599. doi: 10.1016/j.tics.2017.05.002 28578977 10.1016/j.tics.2017.05.002

