# Supplementary figures and images for: Examining the Triple Code Model in numerical cognition: An fMRI study
Source: PLoS One. 2018 Jun 28;13(6):e0199247. doi: 10.1371/journal.pone.0199247 (PMC6023115; doi:10.1371/journal.pone.0199247)

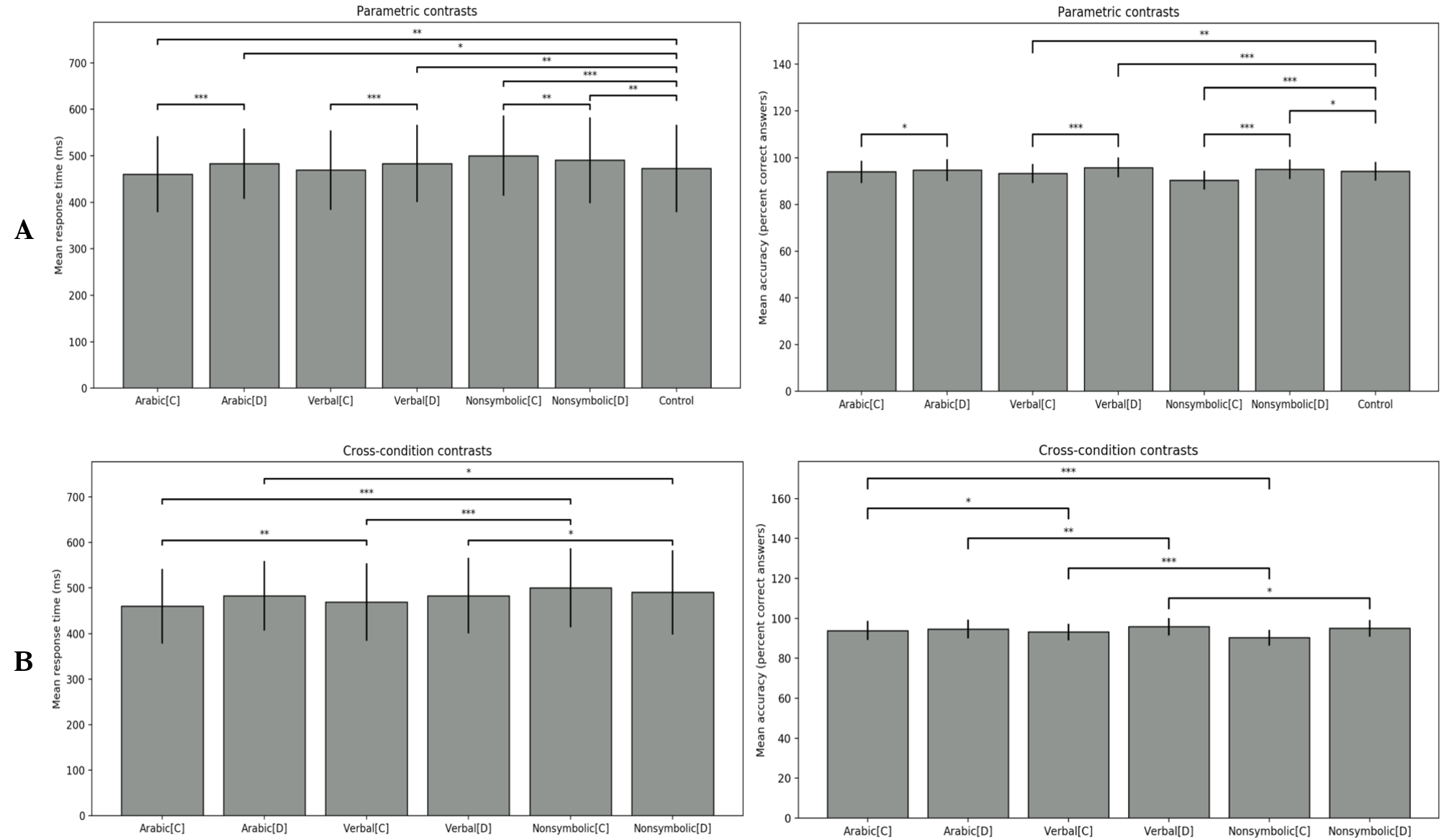

Supplement: S1 Fig — Significant differences in reaction times and response accuracy are illustrated across tasks. Close and distant numerical trials are indicated by [C] and [D] respectively. (A) Parametric contrasts of response time (left) and accuracy (right). (B) Cross-condition contrasts of response time (left) and accuracy (right). (TIF) [file pone.0199247.s001.tif]
